# Integrating AI and Molecular Modeling for Structural Prediction of a Closed-Pore State of the hERG Channel

**DOI:** 10.1021/acs.jcim.6c01219

**Published:** 2026-08-10

**Authors:** Catherine Upex, Thomas Osborne, Giovanni Biglino, Jules C. Hancox, Robin A. Corey

**Affiliations:** † Bristol Medical School (THS), Level 7, Bristol Royal Infirmary, Upper Maudlin Street, Bristol BS2 8HW, United Kingdom; ‡ Bristol Medical School (THS), Cardiovascular Research Laboratories, Biomedical Sciences Building, 1980 University Walk, Bristol BS2 8HW, United Kingdom; § School of Psychology and Neuroscience, Biomedical Sciences Building, University Walk, Bristol BS8 1TD, United Kingdom

## Abstract

The voltage-gated potassium channel hERG (Kv11.1) plays a central role in cardiac repolarisation by mediating the rapid delayed rectifier K^+^ current (*I*_Kr_). Blockage of hERG by small molecules can lead to delayed repolarisation, QT interval prolongation, and potentially fatal arrhythmias, making the channel a critical focus in drug safety screening. Despite extensive pharmacological and electrophysiological characterization, a complete structural understanding of hERG gating remains limited by the absence of an experimentally determined closed-state structure. Here, we use AI-based structural modeling to predict and compare candidate closed conformations of hERG. Building on recent work in which AlphaFold2 (AF2) predictions guided by engineered structural templates captured closed and inactivated states, we applied the emerging protein structure predictor, Chai-1, which employs a single-sequence, language model-based approach independent of multiple-sequence alignments (MSAs). The resulting Chai-1 hERG model was compared with the AF2-derived closed structure, a homology model based on the *Rattus norvegicus* EAG channel, and an experimentally resolved open-state cryo-EM structure. We assessed these models using a combination of all-atom and coarse-grained molecular dynamics simulations, analyzing protein dynamics, pore geometry, gating residue orientation, hydration, and lipid interactions. The Chai-1 and AF2 models displayed strong structural and dynamic agreement, both adopting compact, non-conductive conformations consistent with a physiologically closed-pore state. When run using an MSA, the Chai-1 predictions sample additional conformations with a distinct positioning of the voltage sensing domain (VSD) gating residues. Our molecular dynamics data reveal insights into pore and VSD dynamics, and support a previously identified role for ceramide binding at the M651 residue. Our findings support the plausibility of AI-derived closed-pore state hERG models and underscore the growing potential of deep learning-based protein structure prediction to identify previously uncharacterized, pharmacologically relevant conformations of membrane proteins. Further, our Chai-1 derived closed-pore state model expands our structural insights into hERG gating and may have utility for investigation of drug-hERG interactions.

## Graphical abstract

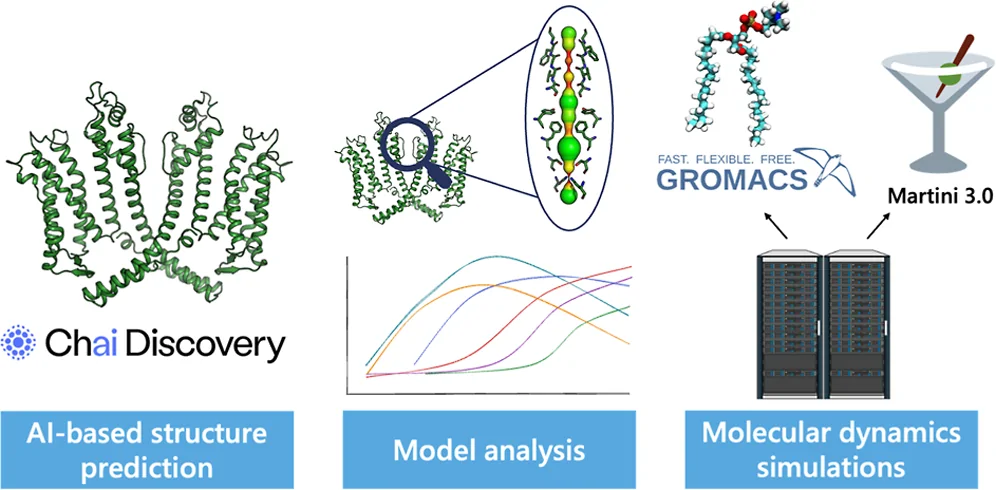

## Introduction

The voltage-sensitive potassium (K^+^) channel Kv11.1, also known as hERG1 (hereafter hERG), plays a critical role in the repolarisation phase of the cardiac action potential by mediating the rapid component of the delayed rectifier K^+^ current (*I*_Kr_).^1^ Owing to this essential physiological role, perturbations to hERG function can have serious clinical consequences. hERG is notably promiscuous in its pharmacology; blockade of the channel by small molecules leads to delayed repolarisation of the cardiac action potential, manifesting as QT interval prolongation on an electrocardiogram—a condition referred to as drug-induced long QT syndrome (diLQTS)—which can precipitate fatal cardiac arrhythmias.^2–4^ Consequently, understanding how novel compounds interact with hERG is a critical aspect of early drug discovery and safety evaluation.

Despite extensive interest in hERG as a pharmacological target and antitarget, structural information on the channel across its full conformational landscape (open, closed, and inactivated states) remains incomplete. Several open-state structures have been resolved using cryo-electron microscopy (cryo-EM), including complexes with high-affinity blockers.^5–7^ However, we note that none of these structures have been conclusively demonstrated to be conductive in molecular dynamics (MD) simulations, so arguably could be considered “open-like”. In addition to the open/closed states, hERG channels undergo rapid C-type inactivation, a process thought to involve asymmetrical rearrangements within the selectivity filter rather than closure of the intracellular activation gate,^8^ and this non-conductive state—interpreted as inactivated—has recently been elucidated by exploiting the channel’s K^+^-sensitive inactivation mechanism.^6^ An experimentally derived closed-state structure, however, remains elusive, with current best practice using homology modeling based on the closely related *Rattus norvegicus* EAG channel (PDB: 5K7L^9^),^10^ or derived using a MD-relaxed open structure.^11^ The absence of this key conformation limits our understanding of gating transitions and constrains efforts to model state-dependent drug interactions.

The hERG channel exhibits a canonical voltage-gated potassium channel architecture.^6^ It assembles as a tetramer in which each subunit contributes six transmembrane helices (S1–S6). The S1–S4 helices form the voltage-sensing domain (VSD) (Figure 1: yellow), which detects changes in membrane potential via conformational rearrangements. The pore domain is formed by the S5 and S6 helices from each subunit, creating the central ion conduction pathway. Within this region, the S6 helix (Figure 1: green) lines the inner pore and plays a key role in gating, while the selectivity filter (Figure 1: pink), located at the extracellular end of the pore, confers potassium ion selectivity. Finally, a C-linker (Figure 1: cyan) couples the cyclic nucleotide-binding homology domain (CNBHD: not present in structure) to the pore. Together, these structural elements coordinate to underpin the distinctive gating kinetics and ion conduction properties of hERG.

**Figure 1:**
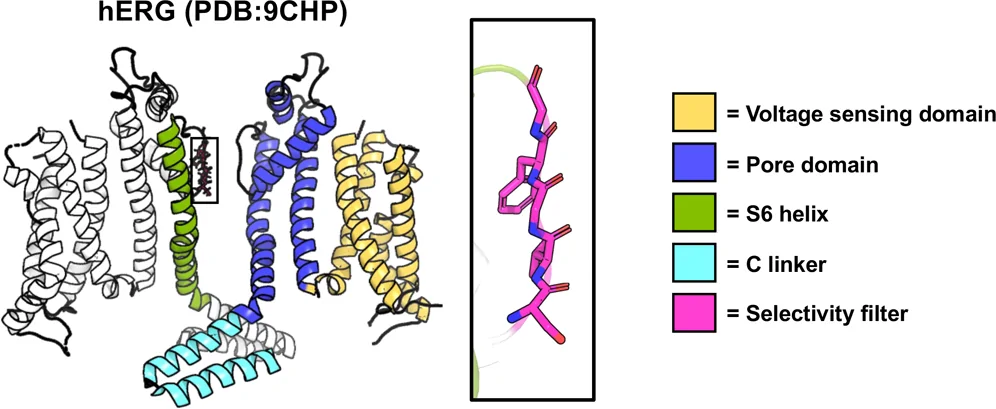
Structural architecture of the hERG channel. Shown are two subunits from the cryo-EM structure of hERG determined in 300 mM K^+^ (PDB: 9CHP). Key regions are highlighted, including the voltage-sensing domain (yellow), C-linker (cyan), and pore domain (blue). The pore domain contains the S6 helix (green) and the selectivity filter (pink).

Advances in computational protein structure prediction have provided new avenues for addressing the gaps in our current structural understanding of the hERG channel. AlphaFold2 (AF2), a deep learning model that integrates multiple-sequence alignments (MSAs) to predict 3D protein structures, has been transformative in structural biology, often achieving near-experimental accuracy even in the absence of close homologues. AF2 and other AI-based modeling methods have been applied to look at a range of protein targets, including a range of voltage-gated ion channels.^12,13^ Recent work using AF2 with engineered structural templates led to production of putative closed and inactivated hERG conformations.^14,15^ For this, the authors went beyond a “blind” AF2 prediction: they engineered structural templates to guide the network toward functionally relevant conformations, effectively steering it to sample gating-relevant structural states that pure homology or MSA-based prediction might miss. Their models exhibited plausible pore dimensions, key gating residue orientations, and features consistent with known mutational and electrophysiological effects, lending confidence that these in silico states may approximate true physiological conformations.^14,15^ Another AI-based modeling program, Chai-1, offers an alternative approach that can operate in single-sequence mode, drawing on large-scale protein language models to infer structural features without reliance on MSA.^16^ AF2 and Chai-1 are different AI models with different architectures and training data, meaning that for any given protein they might predict a slightly different structure. To the best of our knowledge, Chai-1 has not yet been applied to modeling any human ion channels, aside from our own recent work on modeling synthetic cannabinoid receptor agonists (SCRAs).^17^

Given the importance of accurate closed-state models for pharmacological screening, we sought to independently support and extend these recent AF2-based predictions using an alternative AI-driven framework. We generated a closed-pore-state hERG model using Chai-1 in an “out-of-the-box” manner (i.e., generating 5 models using the default settings), and compared it to the AF2-derived closed model, a homology model built for this study based on the *R. norvegicus* EAG channel (PDB: 5K7L), and two experimentally resolved structures, representing the open (PDB: 9CHP) and non-conductive or inactivated state (PDB: 9CHQ). We assessed these models using a combination of structural and dynamic metrics, including pore geometry, helix dynamics, water and ion permeation (via all-atom molecular dynamics), and lipid interactions (via coarse-grained molecular dynamics).

Our findings reveal a high degree of structural similarity between the Chai-1 and AF2 closed-state models, providing supporting evidence of both as plausible representations of the channel’s closed conformation. These results further highlight the growing potential of AI-based modeling to identify previously uncharacterized, pharmacologically relevant states of membrane proteins,^18–22^ thereby enhancing both mechanistic understanding and predictive drug-safety evaluation.

## Results

### Use of AI-Based Modeling to Generate a Closed-Pore Model of the hERG Channel

All five Chai-1 hERG models exhibit consistently high confidence, particularly within the core region of the channel. All models show strong interchain predicted TM-scores (ipTM ≈0.83; see Table S1), indicating reliable complex assembly, while the predicted local accuracy (pLDDT) is especially high throughout the complex, especially the pore region (Figure S1 and Table S1), supporting the structural integrity of the key functional domains. Structurally, the Chai-1 models closely resemble the previously published template-based AF2 model,^14^ with particularly strong agreement in the central pore architecture. Although some variability is observed in VSD and C2 linker, the pore domains remain highly conserved across models, with root-mean-square deviations (RMSDs) of approximately 0.53–0.57 Å (Figure S2). Notably, this agreement is slightly better than that observed with the *R. norvegicus* cryo-EM EAG closed state structure (PDB: 5K7L^9^), which is commonly used^10^ as a template for human hERG modeling, where RMSDs range from ∼0.63 to 0.86 Å (Figure S3). Additionally, the Chai-1 models are distant from the experimentally derived open structure (PDB: 9CHP), where RMSDs sit at ∼20 Å (Figure S4). Given the high ipTM and pLDDT scores across all models, we selected model 2—the model with the closest RMSD to the EAG closed structure (Figure S3; Table S1)—to take forward for downstream analysis (Figures 2A and S5).

**Figure 2:**
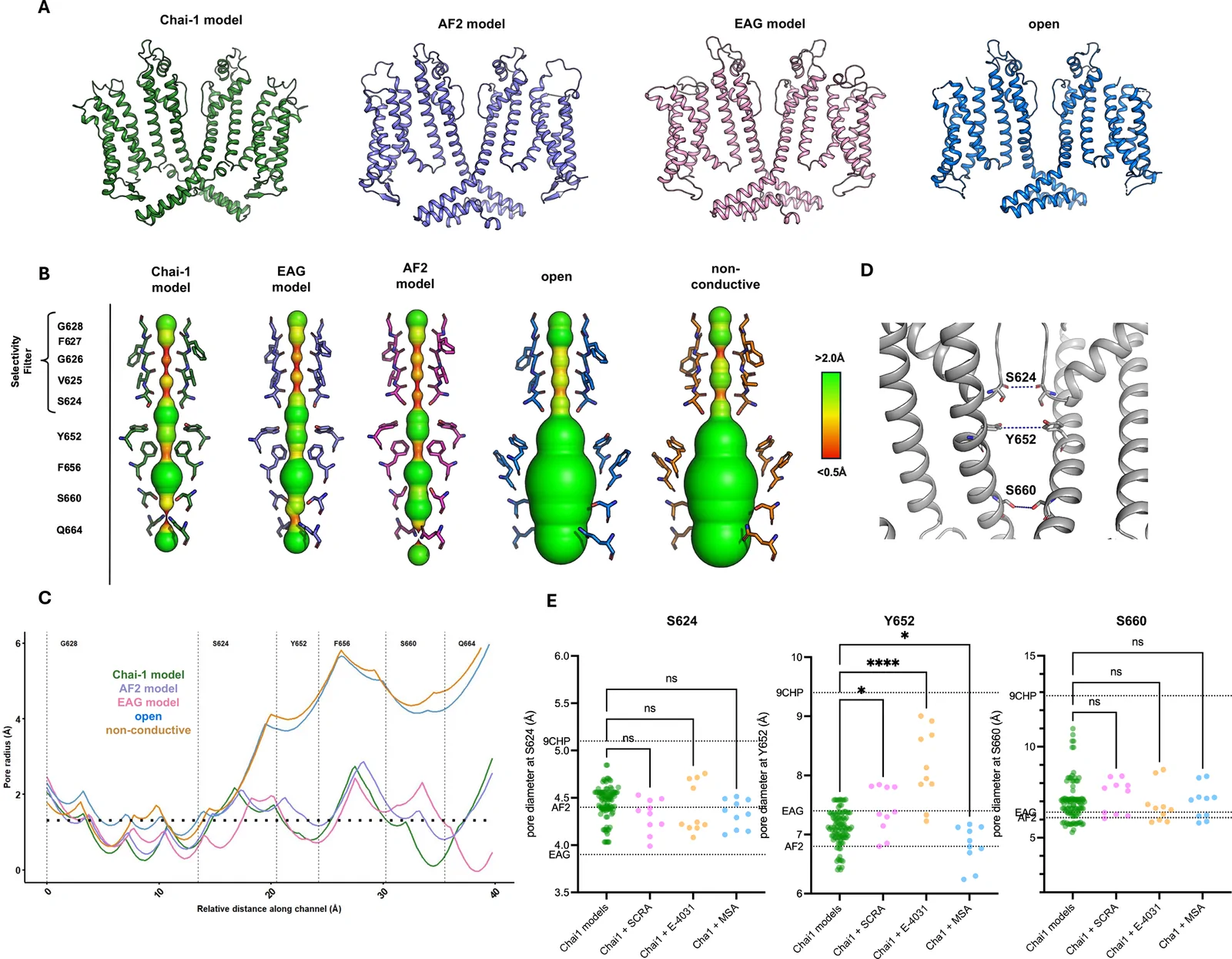
Comparison of Chai-1 models with previously reported hERG structures. (A) Structural alignment of Chai-1 model 2 with the previously published template-based AF2 model (“AF2 model”), a homology model based on the EAG structure (“EAG model”: based on PDB 5K7L), and the open-state cryo-EM structure (“open”: PDB 9CHP). (B) Close-up view of key pore-lining residues across all models, shown in the context of their corresponding HOLE profiles, with the addition of a non-conductive cryo-EM structure for comparison. (C) Pore radius profiles calculated using HOLE for the structures shown in panel B. (D) Central region of the hERG channel showing the residues used for cross-pore distance analysis, displayed as stick representations. (E) Distances between residue pairs shown in panel D, measured between diagonally opposing hERG subunits. Reference distances from the input models are indicated by dotted lines. Two values are shown for each Chai-1 model, corresponding to measurements between chains A-C and B-D. Statistical significance was assessed using one-way ANOVA, followed by Dunnett’s multiple-comparisons test, in relation to the Chai-1 data.

### Pore Analysis of the Chai-1 Closed-Pore Model

The selected Chai-1 model exhibits a pore profile that provides computational support for a closed-pore state across key gating regions (Figure 2B). In the selectivity filter region (around G628-S624), the pore radius in the Chai-1 model drops below ∼1.5 Å, approaching or falling beneath the approximate radius required for K^+^ permeation (indicated by the dashed line). This suggests that the filter adopts a constricted conformation that limits K^+^ passage. Comparison to the closed AF2 model and the closed EAG-based homology model suggests a very high degree of similarity in this region, including in the orientation of key selectivity filter residues (Figure 2C) supporting its designation as a closed-pore state. The open and non-conductive cryo-EM structures are generally wider in this region, reaching above 1.5 Å.

Moving into the central cavity (around the canonical binding residues: Y652–F656), the Chai-1 model shows a moderate expansion, with the width increasing to ∼2.5–3 Å, again in line with the AF2 model and EAG-based homology model, and far narrower than the open and non-conductive cryo-EM structures, both of which display substantially wider cavities (>4 Å) (Figure 2C). The side chains of Y652 and F656 closely match those of the AF2 model and EAG homology model, with both pointing into the conduction pathway (Figure 2B). Notably, a constriction in the Chai-1 model occurs toward the intracellular gate region (around S660-Q664). Here, the pore radius sharply decreases to near 0–1 Å, representing a clear steric occlusion (Figure 2C). This is driven by the side chain position of Q664 (Figure 2C). This is substantially narrower than the AF2 model, and mimics a nearby constriction in the EAG-based homology model, also caused by Q664 (Figure 2B).

We next repeated our Chai-1 predictions to generate a further 45 trimmed hERG channel models, making 50 in total. We measured the cross-subunit pore distances for all models at positions S624, Y652, and F656 (Figure 2D), and found that in general a closed pore structure is consistently found, with almost all models being fully closed at S624, and only a very small number moving toward (but not yet at) the open state for Y652 and F656 (Figure 2E). These predictions are not altered substantially by inclusion of a synthetic cannabinoid receptor agonist (SCRA) or known hERG blocker E-4031 in the model prediction, except at position Y652 (Figure 2E). For the E-4031 at least this is likely due to direct interactions between the drug and Y652 residue (Figure S6).

To test the role of MSAs in this process, we reran our predictions using an MSA produced by MMseqs2. The data show the models built using MSAs were more dissimilar to both the AF2 closed model and EAG model (Figures S7 and S8), while still remaining distant from the open model (Figure S9). The pores of the model are similar at positions S624 and S660, but narrower at Y652 than the default Chai-1 models (Figure 2E).

Overall, the Chai-1 pore profile appears to combine a constricted selectivity filter with a tightly closed intracellular gate. This dual constriction is characteristic of a closed state, and despite subtle differences, shows overall a high similarity to the AF2 and EAG homology models in the degree and location of pore narrowing.

### Analysis of the VSD in the Chai-1 Model

Comparison of the voltage-sensing domain (VSD) across models shows that the overall architecture is well preserved, with strong agreement between the Chai-1 models and the previously reported AF2 structure (Figure 3A), and slightly lower similarity to the EAG-based homology model (Figure 3C). The most prominent structural deviation is localized at the C-terminal end of the S4 helix, immediately preceding the S4–S5 linker, a region known to play a critical role in coupling voltage sensing to pore opening.

**Figure 3:**
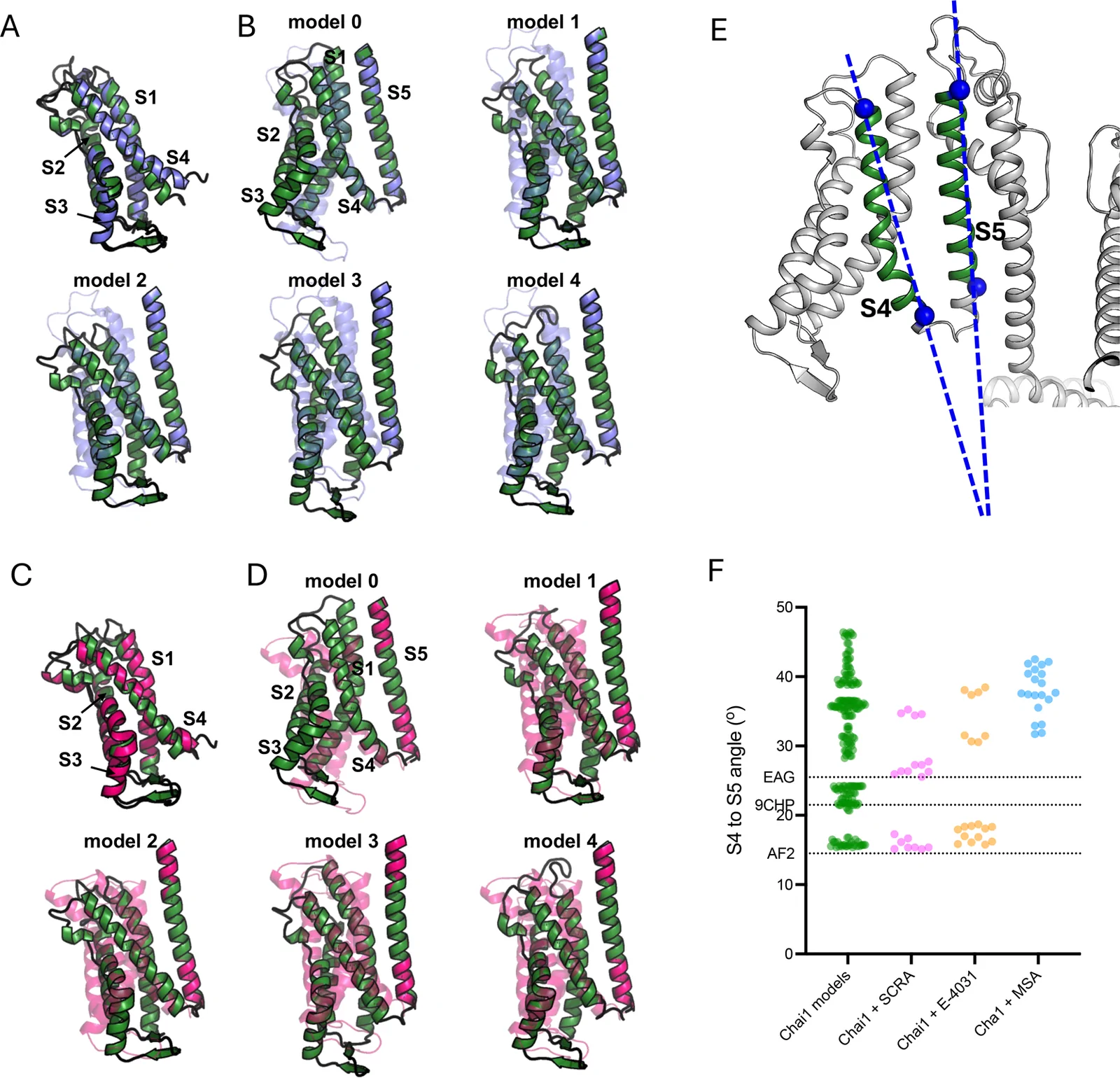
Comparison of the VSD and adjacent S5 helix between models. (A) VSD of selected Chai-1 model (green) aligned to AF2 model VSD (blue), producing an RMSD of 1.1 Å. The other Chai-1 models show similar conservation (1.2, 1.0, 1.0, and 1.1 Å respectively). (B) Views of all five Chai-1 VSDs and adjacent S5 (green) compared to the AF2 model (transparent blue), as aligned on S5. (C) As panel (A) but for the Chai-1 model and the EAG homology model (pink). RMSD = 1.5 Å, and 1.6, 1.5, 1.5, and 1.4 Å for the other Chai-1 models (D) As panel A, but for the EAG homology model (transparent pink). (E) View of the hERG channel showing the residues and helices used to define the S4–S5 angle (F) S4–S5 angles measured for all Chai-1 models. Reference angles from the input models are indicated by dotted lines.

Consistent with this, the relative orientation of the VSD with respect to S5 varies substantially between models. All five Chai-1 models display differences in this interface, indicating a degree of conformational heterogeneity. Notably, Chai-1 model 0 closely recreates the AF2-derived orientation, whereas models 1–4 adopt a markedly different configuration, characterized by a pronounced downward tilt of the VSD relative to the pore domain (Figure 3B). This shift likely reflects alternative positioning of the S4–S5 linker and may correspond to distinct coupling states between the VSD and pore. In contrast, none of the Chai-1 models reproduce the VSD orientation observed in the EAG-based homology model (Figure 3D). To quantify this effect, we measured the angle formed between S4 and S5, modeled using vectors taken between E518 and D540 and between L550 and E575. Running over all 50 produced Chai-1 models reveals a wide range of VSD positions sampled (Figure 3E,F), from an upright position closely resembling the AF2 model (ca. 15°) to a far wider orientation (ca. 45°). Adding either the SCRA or E-4031 produces a similar range of angles (Figure 3F). Interestingly, running models using the MMseqs2 MSA results in only the wider angles being sampled (Figure 3F). Collectively, these observations reinforce the idea that, while the core VSD fold is conserved, its orientation relative to the pore domain is highly variable, with possible functional impacts on channel gating.

As in other voltage gated ion channels, in hERG a series of salt bridges between S2 and S4 are important for gating. Most important are D456, D460, and D466 on S2 and K525, R528, R531, R534, R537, K538 on S4.^23,24^ The acidic residues on S2 flank a central hydrophobic gating residue, F463, with protonation of these residues destabilizing interactions with S4.^25^ In our Chai-1 models, we see a similar positioning of all gating residues in relation to F463, with F463 forming cation–π interactions with both R531 and R534 (Figure 4A). The same stacking is seen in all existing hERG structures, including both open (9CHP^6^ and 5VA1^7^) and inactivated (9CHQ^6^) structures, as well as in the published closed AF2 model and the closed EAG structure (Figure 4A). The AF2 study identified a kink in the S4 helix that is also present in EAG model. We do not see this in our Chai-1 models but this does not seem to impact residue positioning (Figure 4A).

**Figure 4:**
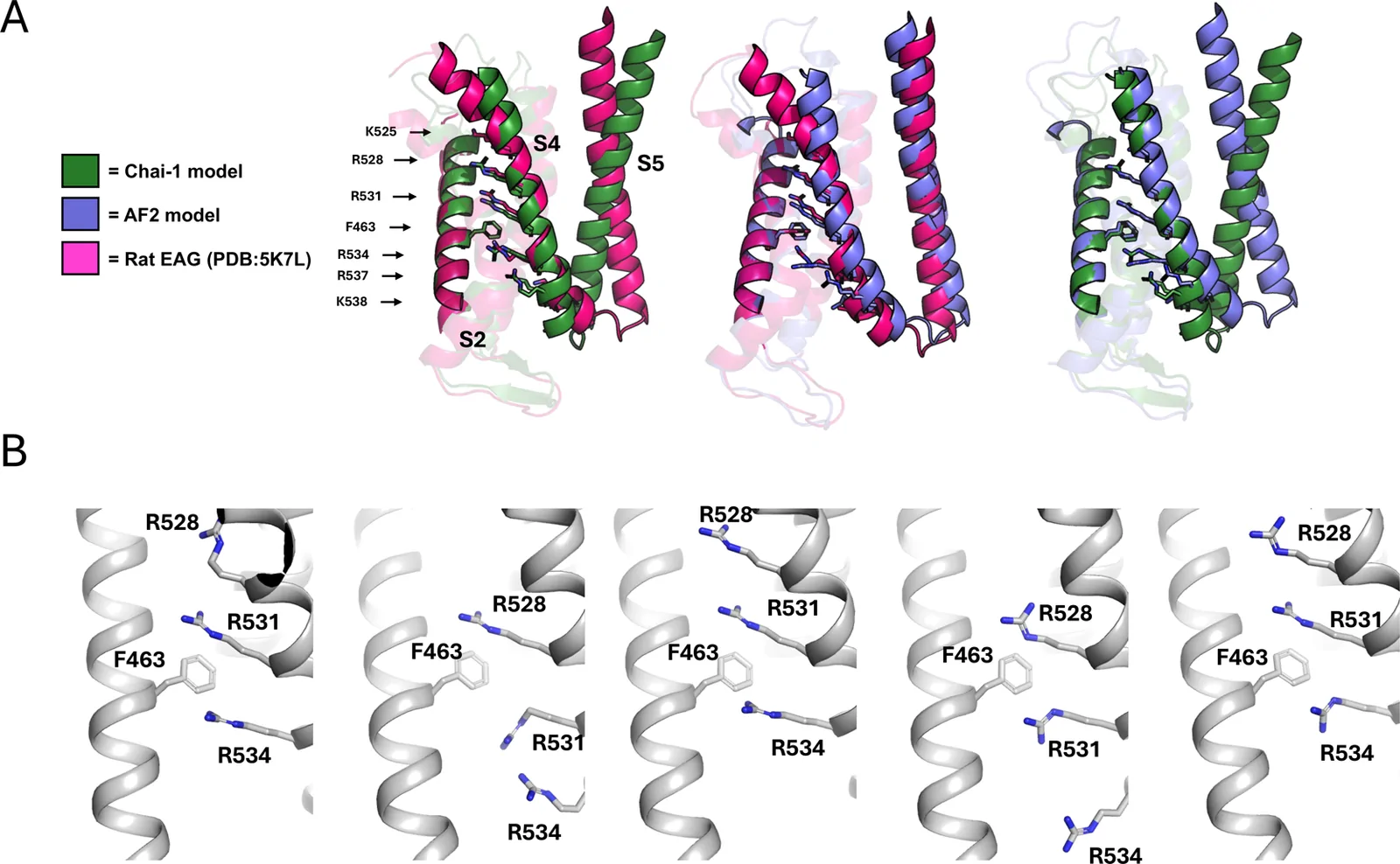
View of VSD gating residues from hERG models (A) Comparison of the positions of key VSD gating residues in the Chai-1 model with previously reported hERG models and structures. The central hydrophobic charge-transfer residue F463 and the positively charged S4 gating residues are shown as stick representations. (B) Positions of VSD gating residues in five Chai-1 models generated using the MMseqs2-derived MSA. Shown are F463 and the three nearest positively charged gating residues on S4, highlighting variation in the relative positioning of the gating-charge network between models.

Of note, running the calculations using the MMseqs2 MSA provides more variation in this region, with 2 out of 5 models positioning F463 between R528 and R531 (Figure 4B), whereby the S4 helix has moved downward by one gating-charge register relative to the existing cryo-EM structures and models. These models are therefore part way toward the theorized hyperpolarised down state, where F463 sits between K525 and R528.^26^

### Stability and Dynamics of the hERG Models

We next performed atomistic MD simulations to assess the stability and dynamics of the Chai-1 model, comparing it against the AF2 model, the EAG-based homology model, and the open cryo-EM structure. For each system, we ran five independent 1 μs replicates in a fully solvated lipid membrane. Root-mean-square deviation (RMSD) analysis of the protein backbone revealed that while all hERG models were generally stable, the two experimental structures exhibited greater overall stability than the Chai-1 and AF2 predictions (Figure S10A) This was especially the case for one replicates of each system which showed higher dynamics (>0.6 nm RMSD) (Figure S10A; magenta traces).

Root-mean-square fluctuation (RMSF) analysis indicated that the core motifs of the hERG channel remained generally stable across all simulations (Figure 5A). Notably, the S2–S3 linker in the open state was more dynamic than in the closed states, whereas all three closed states exhibited higher flexibility in the S3 helices compared to the open state. The EAG-based model showed a significantly more dynamic S4 helix than all other states, while the open state displayed a more dynamic S5. The turret helix was consistently dynamic across all states, however, the pore helix was markedly stabilized in the open state relative to the closed states. In all systems, the C-linker (C2) was the most dynamic region (Figure 5A). However, in the full length hERG model, the C-linker is likely to form interactions with regions of the channel, especially the CNBHD, that are missing in our models. Accordingly, rerunning RMSD analyses with the C2 region removed reduces the RMSD, especially for the previously high repeats for AI-based models (Figure S10B). Modeling of monomeric full-length hERG can reintroduce these interactions, although with a drop in model quality (Figure S11).

**Figure 5:**
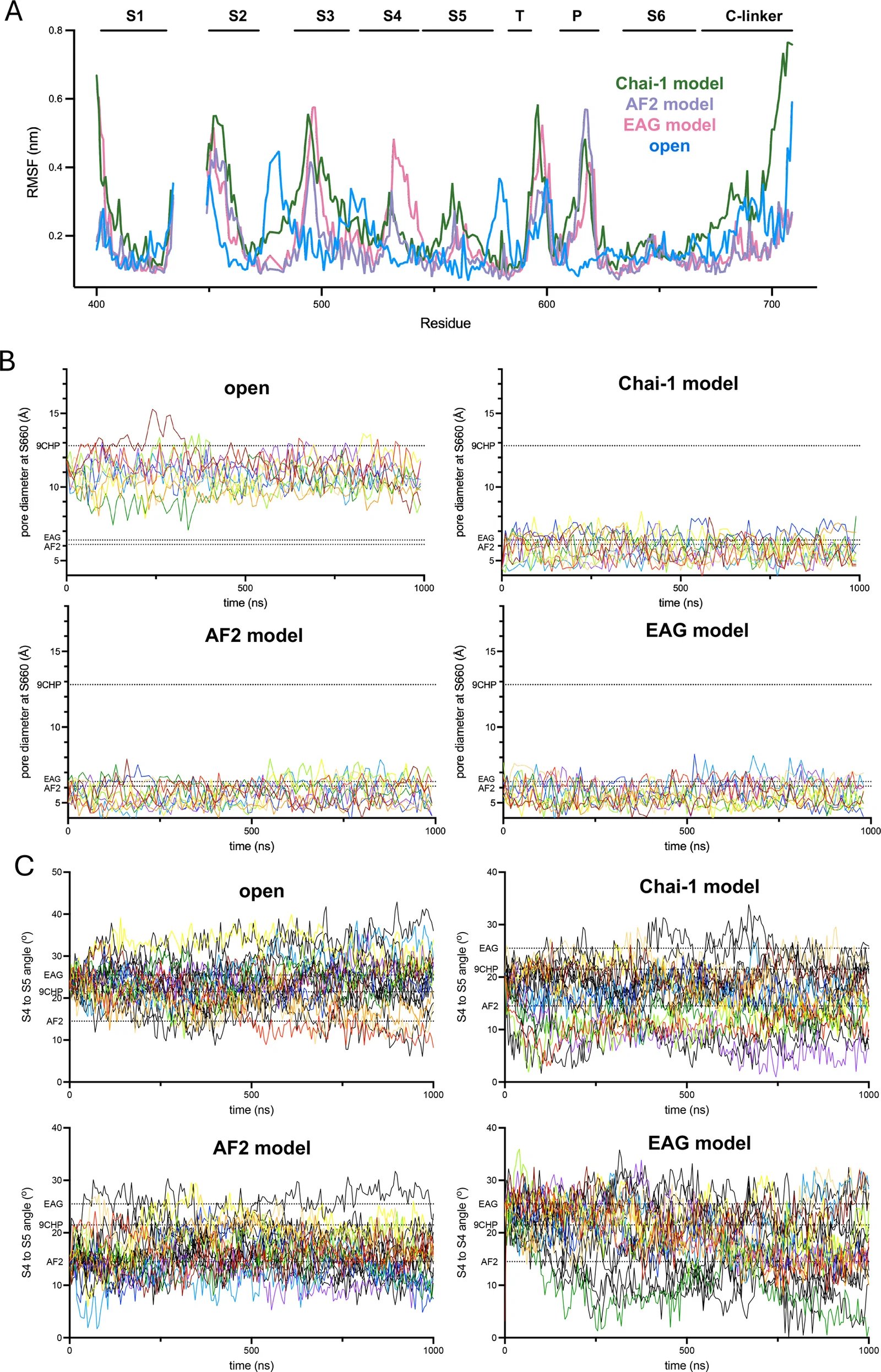
Molecular dynamics of the hERG models. (A) Per-residue RMSF analysis along hERG, averaged across all four subunits and across 5 independent 1 μs simulations. S1–6 helices are labeled, along with the turret (T) and pore (P) helices and C-linker. (B) Distances between Ser660 residues from opposing subunits are plotted over time during 5 × 1 μs atomistic MD simulations for the open structure, and Chai-1, AF2 closed, and EAG models Two distances are shown for each system, corresponding to chains (A–C) and (B–D). Equivalent analyses for residues S624 and Y652 are presented in Figures S12 and S13. Distances measured in the input models are indicated by dotted lines. (C) S4–S5 angles are plotted over time during 5 × 1 μs atomistic MD simulations (see Figure 3E for angle definition). Four measurements are shown for each system, corresponding to the four channel subunits. Angles measured in the input models are indicated by dotted lines.

We then used the same pore distance and S4–S5 angle measurements defined in Figures 2E,F and Figure 3F to analyze the MD trajectories. The data reveal relatively stable pore dimensions at the intracellular gate (S660) for all models (Figure 5B). A similar trend was observed for S624 (Figure S12), although with somewhat greater variability, with several open-state replicates exhibiting pore widening and one EAG replicate undergoing substantial narrowing. Distances involving Y652 were generally more dynamic, particularly in the open-state simulations (Figure S13), consistent with the increased flexibility of the central cavity region.

In contrast to the pore measurements, the S4–S5 angles between the pore and VSD exhibited substantially greater conformational variability throughout the simulations (Figure 5C). All models sampled a broad range of angles, suggesting that the VSD-pore interface remains dynamic. The EAG model in particular deviated considerably from its starting conformation, although variation is seen in all systems. This matches the wider range of values seen for the input Chai-1 models (Figure 3F). Together, these observations suggest that the VSD-pore interface is intrinsically more dynamic than the pore itself, allowing substantial variation in S4–S5 geometry while maintaining a relatively stable pore architecture. Therefore, predicted interactions at the VSD-pore interface, whether from AI models, structural data, or homology modeling, are likely to be more variable than those in the pore.

### Analysis of the hERG Solvation during MD

Next, we analyzed the channels using the CHAP package.^27^ This analysis revealed marked differences in pore hydration between the open and closed conformations. The open-state model remained continuously hydrated, exhibiting no significant free-energy barrier to water occupancy at the center of the channel (Figure 6A). In contrast, the Chai-1 model displayed a pronounced barrier of approximately 2.5 kT, indicative of partial dewetting within the central pore Figure 6A. A similar barrier was observed for the AF2-derived closed model (Figure S14A). These findings were corroborated by water density maps generated using the VolMap tool in VMD (Figure S14B) and by CHAP solvent density profiles (Figure S14C). Together, these analyses demonstrate that, unlike the open structure, the Chai-1, AF2, and EAG models all adopt pore conformations that are unfavorable for water permeation, consistent with non-conductive channel states.

**Figure 6:**
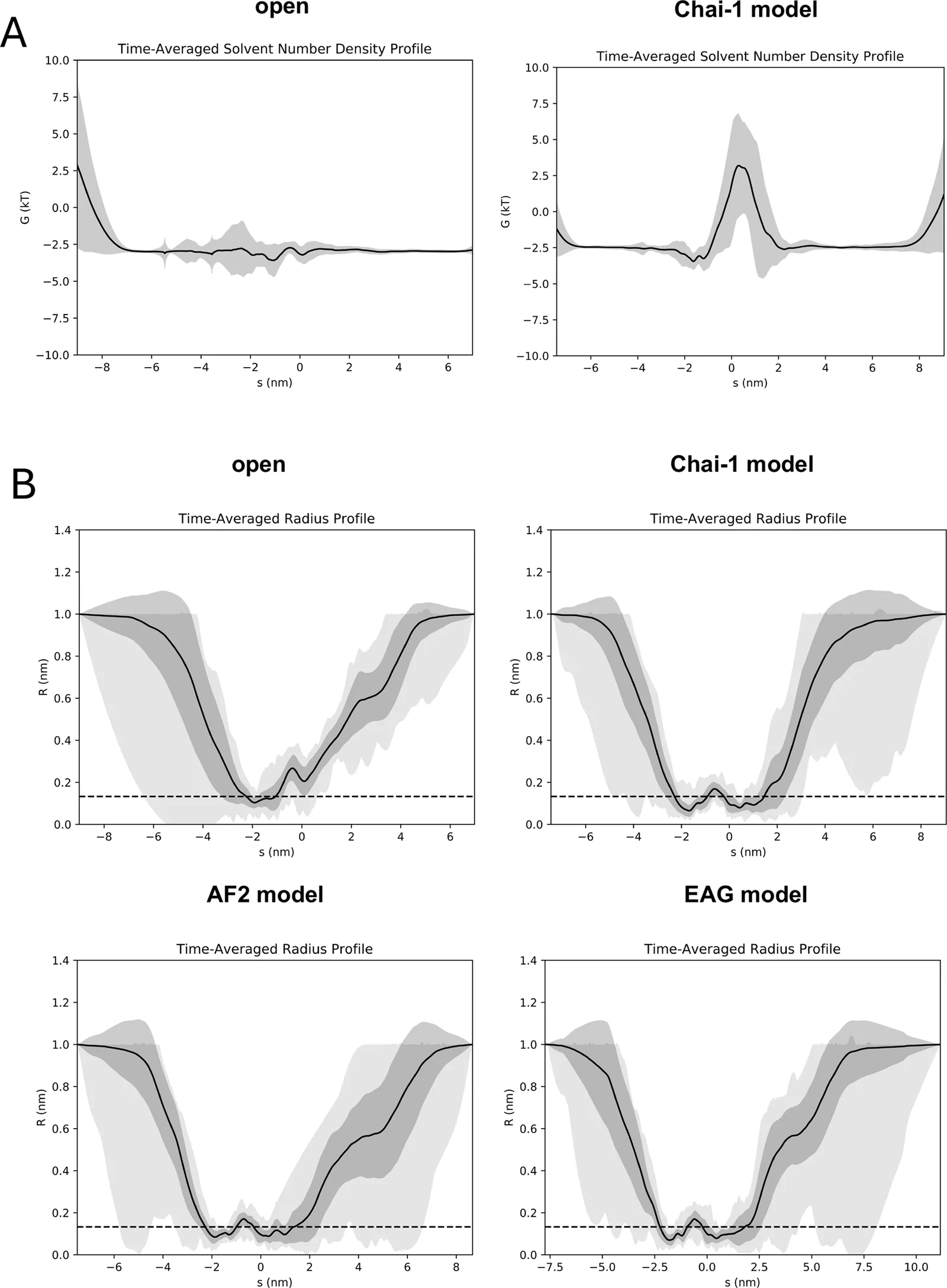
CHAP analysis of hERG channels: (A) time-averaged solvent density profiles through the hERG channel over 5 × 1 μs atomistic MD simulations, expressed as solvation free energy relative to kT and plotted along the channel axis (s), as calculated using CHAP. The gray shading represents the standard deviation across simulation frames. (B) Time-averaged pore radius profiles of the hERG channel over 5 × 1 μs atomistic MD simulations, calculated using CHAP. Dark gray shading indicates the standard deviation across simulation frames, while light gray shading indicates the full range of observed values.

Analysis of pore geometry revealed that the overall pore dimensions remained stable throughout the simulations and closely resembled those of the initial input structures. The open-state model maintained a minimum pore radius close to the ionic radius of K^+^ (1.33 Å), supporting a hydrated permeation pathway throughout the simulation (Figure 6B). In contrast, all three closed-state models exhibited two pronounced constriction sites with radii substantially below this threshold. These constrictions coincided with the regions of reduced hydration identified by CHAP, further supporting the assignment of these structures as closed, non-conductive conformations.

Notably, despite differences in overall architecture, the three closed-state models converged on similar pore-radius and hydration profiles, suggesting that pore closure imposes common structural constraints irrespective of the modeling methodology used to generate the starting structures.

### Lipid Binding Sites in the Different hERG States

A previous study^28^ used MD to probe lipid interactions with the hERG channel using an open hERG model.^7^ We reasoned that it would be useful to re-evaluate these interactions using the two recent closed-pore state predictions (our Chai-1 and the previously published AF2) and the newer open-state cryo-EM structure (PDB: 9CHP^6^). Furthermore, whereas previous work employed the Martini 2.2 force field,^29^ we utilized the updated Martini 3.0.^30^

All models were subjected to five independent 10 μs coarse-grained MD simulations in a mixed lipid bilayer containing 20% ceramide (Martini lipid “DPCE”) and 5% of the polyunsaturated lipid (C18:2) dilinoleoylphosphatidylcholine (DIPC; Martini “DIPC”). Previous work identified M651 as a key residue involved in ceramide binding, so our initial analysis focused on this site. Across simulations, we observed substantial enrichment of ceramide in the vicinity of the channel, as evidenced by both lipid occupancy analysis using the PyLipID package^31^ and spatial density maps calculated in VMD (Figure S15).

Binding duration analysis of lipid interactions using PyLipID revealed significant enrichment of ceramide around M651-containing binding sites in all models (Figure 7A), consistent with previous findings.^28^ Sites involving M651 generally exhibited longer lipid residence times than other identified interaction sites (Figure 7B). Analysis of the highest-occupancy binding poses showed direct contacts between ceramide molecules and the M651 side chain (Figure 7C), further supporting a key role for this residue in mediating ceramide interactions with hERG.

**Figure 7:**
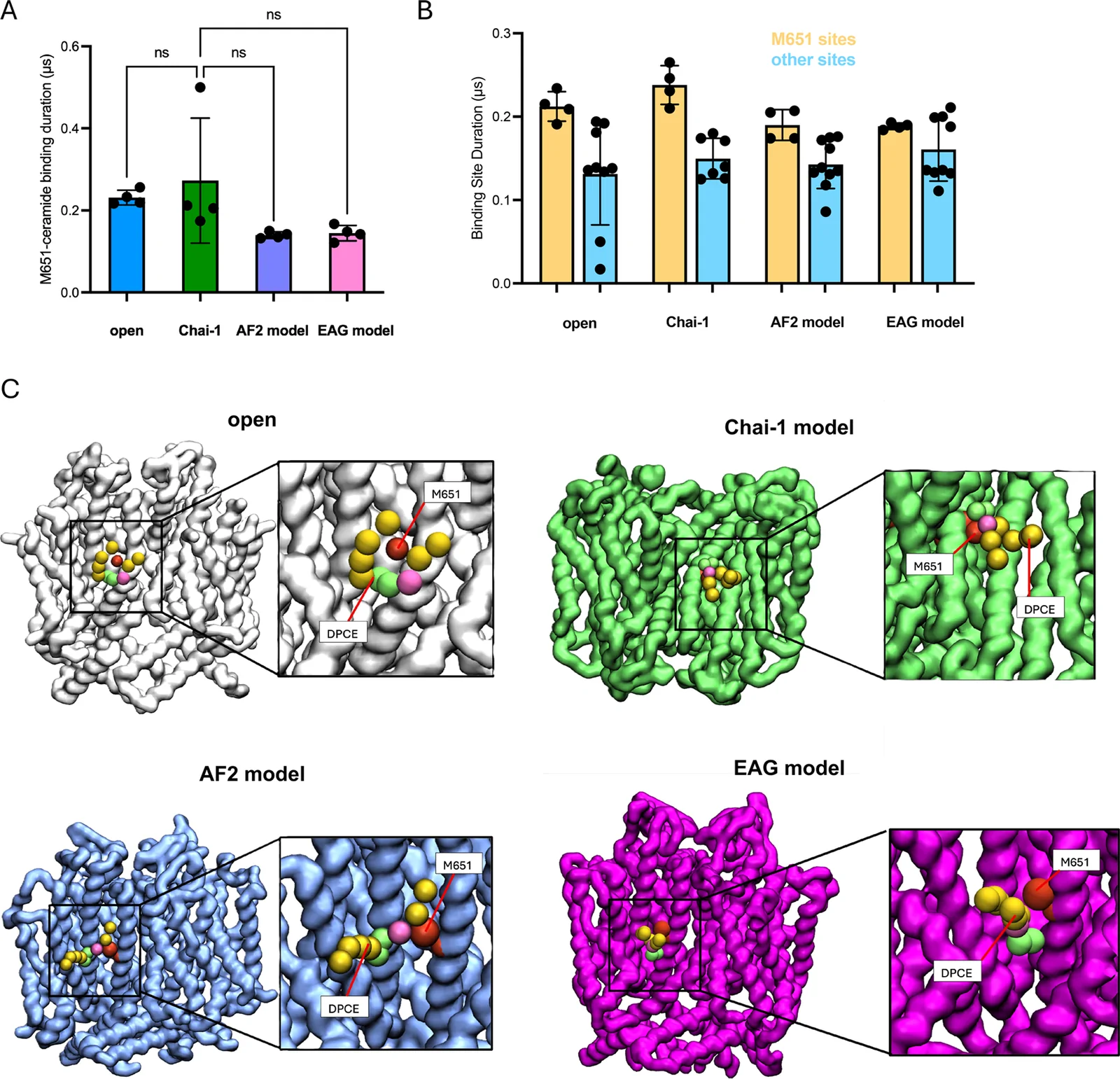
Coarse-grained MD and lipid interactions. (A) PyLipID Quantification of binding duration of ceramide at residue M651 for each of the hERG channel models. Statistical significance was assessed using one-way ANOVA, followed by Dunnet’s multiple-comparisons test, in relation to the Chai-1 data.. (B) Plot showing ceramide duration at binding sites either containing M651 or not, as quantified using the PyLipID package (C) PyLipID-derived top poses of ceramide binding at a representative M651-containing site.

## Discussion

Ion channels, such as hERG, do not exist in a single static conformation but instead occupy a continuum of interconverting microstates—indeed, this underpins their complex gating behavior. As such, no single structure can fully capture the functional landscape of the channel, and the availability of multiple structurally and dynamically distinct models provides a more comprehensive framework for understanding ion channel function. In this area, AI-based modeling tools can be of considerable value. AF2 has previously been used to explore Na*v* channel conformational states, finding that the output models represent multiple channel conformations, including both experimentally observed and novel conformations.^13^ Another study comparing AF2 with RoseTTAFold^32^ and ESMfold^33^ suggest these methods capture the voltage-sensing and pore domains of voltage-gated ion channels with high confidence and in a number of states, although they highlight uncertainties of modeling unstructured loop and termini regions.^12^ Finally, a template-based AF2 modeling study of hERG demonstrated their approach was able to predict a range of experimentally unresolved hERG states, providing insights into hERG inactivation mechanisms and uncovering molecular features relating to drug binding.^14^

Our results highlight the utility of a different AI-based modeling package, Chai-1,^34^ as another powerful tool for predicting functionally relevant conformational states of ion channels, particularly in cases where such states are not readily captured by default structure prediction pipelines. Our Chai-1 derived hERG model adopts a closed pore architecture, as evidenced by the pronounced constriction at the intracellular gate and the overall HOLE profile. This is notable because standard AF2 predictions typically favor more open or intermediate conformations, and obtaining a fully closed hERG structure typically requires extensive intervention, including template biasing, multiple sequence curation, or iterative refinement workflows. In contrast, Chai-1 appears capable of natively sampling this important closed-pore state without the need for such highly involved pipelines. This commends the utility of AI-based modeling, where previously modeling of the hERG closed state required extensive and complex modeling pipelines.^35,36^

Importantly, while the Chai-1 model shows strong overall agreement with both the AF2-derived and EAG-based homology models, the subtle but consistent differences in pore geometry—particularly within the central cavity and intracellular gate—may reflect a distinct conformational state. Given the well-established state dependence of hERG drug binding, these variations could have meaningful implications for drug interactions, especially for compounds that preferentially bind to the closed state. As such, our Chai-1 structural model expands the available structural repertoire for hERG, offering an additional and potentially more physiologically relevant template for future structure-based drug design and in silico screening efforts.

### Structural Changes within the Pore and VSD

From a mechanistic perspective, differences observed within the voltage-sensing domain (VSD) are of particular interest in the context of channel activation. AI structural modeling represents a powerful method for generating insights into the dynamics of this region, as demonstrated in a recent study using AF2 to produce a large number of VSD structural states, including activated, deactivated, and intermediate conformations.^37^

Our Chai-1 modeling recapitulates the overall architecture of the VSD observed in both experimental structures and AF2-based models, with strong structural conservation of the key charged residues. In particular, we see the S2 central hydrophobic gating residue F463 sandwiched between R531 and R534, in a so-called “up” conformation,^26^ with this same conformation seen in experimentally derived open or activated structures.^38^ Therefore, the VSD in our default Chai-1 models appears most consistent with an activated or “up” conformation, as is also seen in the closed AF2 and closed EAG models. Of note, previous homology modeling on hERG has suggested that, during hyperpolarisation, S4 shifts into a distinct “down” conformation where R531 sits below F463.^26^ When our Chai-1 modeling was done with an MSA, both “up” and “down” conformations are observed (Figure 4B), suggesting that a wider variety of VSD conformation can be achieved through supplementation of Chai-1 with MSAs.

Additionally, the relative orientation of the domain shows notable variation (Figure 3). The data indicate that, while the global VSD fold is conserved, shifts in orientation may reflect alternative conformational states or coupling relationships between voltage sensing and pore gating. In particular, we observe substantial movement of S4 relative to S5, driven by rearrangements in the S4–S5 linker. Given that the S4 helix serves as the primary voltage sensor, undergoing outward and rotational motion in response to membrane depolarisation,^39^ such variability is likely to have direct implications for channel activation. The S4–S5 linker is well established as a key mechanical transducer that couples VSD motion to opening of the intracellular gate,^40^ and subtle differences in its conformation may therefore bias the channel toward distinct gating states. Importantly, despite these differences in orientation, the overall architecture of the VSD-pore interface remains relatively conserved across the Chai-1, AF2, EAG, and experimental structures, supporting the physical plausibility of the predicted coupling arrangements. Interactions within S4–S5 interface between aliphatic side chains have been suggested to stabilize the open state, preventing the downward movement of S4 toward the cytoplasmic side of the membrane.^41^ A mutation within this interface (L532P) results in an increased rate of channel deactivation, potentially the result of reduced S4–S5 coupling and stability.^41^ In this regard, the structural plasticity of this region in our Chai-1 models, along with the increased flexibility in the MD in the EAG-based model (Figure 5) may reflect functionally relevant heterogeneity within this coupling interface, where destabilization of the S4–S5 interface may be reflective of an “nonopen” conformation.^41^ However, we note that the pLDDT of these regions is lower than the central pore, raising the possibility that some of the observed variability arises from reduced model confidence. This highlights a broader challenge in interpreting AI-derived structures, namely distinguishing genuine conformational diversity from uncertainty in poorly constrained regions.

An alternative interpretation of our Chai-1 model is that it represents an inactivated rather than a closed state. However, C-type inactivation in hERG is generally associated with asymmetric conformational changes in the selectivity filter,^8^ whereas the most prominent feature of the Chai-1 model is constriction of the intracellular pore. Furthermore, the selectivity filter architecture is largely preserved relative to experimentally determined hERG structures, suggesting a closed-pore state rather than an inactivated conformation.

Additional differences in dynamics are observed in MD simulations within regions implicated in VSD-pore coupling. The increased mobility of the S2–S3 linker in the open-state model is not straightforward to interpret mechanistically but may influence the energetic landscape of S4 motion, potentially modulating how readily the voltage sensor transitions between states and how effectively these movements are transmitted to the pore domain. This linker has also been proposed to be important for proper transduction of VSD rearrangements to opening and closing the cytoplasmic gate,^42^ so increased flexibility here could reflect a loosening of these interactions in the open state. Conversely, the reduced flexibility of the pore helix in the open state suggests a stabilizing role once the conductive conformation is achieved. A more rigid pore helix may help maintain precise coordination of permeating ions and preserve the geometry of the selectivity filter, both of which are essential for efficient conduction. In contrast, increased flexibility in closed states may reflect a less constrained environment in the absence of ion flux.

### Lipid Binding at the hERG Channel

Miranda et al.^28^ previously investigated the regulation of channel gating by ceramide species, demonstrating that specific sphingolipids can directly modulate hERG function. Using coarse-grained simulations, their analysis focused primarily on the open-state conformation of hERG and identified putative lipid interaction sites centered on M651. Ceramide-mediated modulation of gating kinetics has been demonstrated across the Kv family of potassium channels and are not limited to hERG, with similar effects observed in Kv1.3 and other Kv2 family channels.^43,44^ These effects typically involve VSDs, S4 helices and associated linkers, rather than allosteric gating mechanisms, which are proposed for hERG.^45^

In the present study, we revisited this question using an updated coarse-grained force field,^30^ alongside a more recent open-state hERG model and our newly generated closed-pore state Chai-1 model. Our simulations successfully reproduced the previously reported enrichment of ceramide around M651 and further demonstrated that this interaction is maintained in both open and closed conformations (Figure 7). These findings suggest that M651 represents a conserved lipid interaction hotspot within hERG and indicate that ceramide binding is not restricted to a single gating state. Notably, we have previously identified this same region as a binding site for SCRAs^17^ and the pollutant phenanthrene,^46^ where ligand binding at this site leads to channel block. Together, these observations point to a possible mechanistic link between lipid binding at M651 and modulation of pore gating, with implications for both endogenous regulation and drug-induced hERG inhibition.

### Limitations

While modeling metrics such as pLDDT and pTM scores can be used to assess model confidence, the use of MD provides a more robust cross state and cross method comparison.^47^ Here, we used molecular dynamics (MD) simulations to both test our AI-generated model and probe its dynamic behavior, including protein dynamics, pore structure, water permeation, and lipid interactions. Our data support previous work suggesting that combining AI modeling with MD is a powerful combination for interrogating ion channel structure and function.^14,48,49^ We note that experimental analysis is also of utmost importance for validating newly predicted states, aiming to design experiments that discriminate between these states and known structures.^50^

However, several limitations should be considered. AI-based protein structure prediction methods, including Chai-1 and AF2, are inherently constrained by their training data and may not fully capture the full conformational landscape of highly dynamic systems such as ion channels. This particular study is limited by the current 2048 token limit of Chai-1 meaning that we are unable to model a full length tetrameric channel. This includes several regions of importance, including the CNBHD (residues 734–864). This region is often missing from hERG structures, although has been structurally resolved both as part of the complex using cryo-EM ^7^ and separately using NMR.^51^ However, even if producing a full length tetrameric hERG channel was possible, the low confidence of the hERG Chai-1 single chain models outside of the core region would make interpretation of these structures difficult (Figure S11).

For this study, we selected Chai-1 model 2 as a representative structure. We note that any of the other 49 Chai-1 models could have been selected instead. This selection could potentially impact interpretation of the data, and is an inherent component of the AI-modeling process, where each run will produce a different predicted model.

Here we bolster our AI predictions using MD simulations, but these are limited by accessible time scales and may not adequately sample rare but functionally important transitions. As such, the structures and dynamics presented here should be interpreted as representative snapshots within a broader ensemble. In particular, the combination of AI models and coarse-grained MD adds potential additional uncertainties to the modeling, as inaccuracies in the input structure are less able to be resolved due to the use of elastic networks. Future work might use post atomistic MD snapshots for this, or change the restraints protocol, e.g. to include Go potentials.^52^

With these considerations kept in mind, the Chai-1 model and associated MD data should be viewed as additions to the existing toolkit, complementing experimental structures and other computational models in probing the structural and pharmacological properties of hERG.

### Future and Clinical Outlook

Looking forward, the integration of Chai-1 and related models into drug discovery pipelines offers significant potential. The availability of additional hERG structural states, including the closed-pore conformation described here, expands the toolkit for structure-based screening and may improve early prediction of cardiotoxic liabilities. Given the central role of hERG dysfunction in drug-induced arrhythmias, particularly Long QT syndrome, such advances have the potential to enhance the safety of therapeutic development and, in the longer term, contribute to better outcomes for patients at risk of life-threatening cardiac events.

The future release of a closed-state hERG cryo-EM structure would provide an important benchmark; however, such a structure would still represent only a single conformational snapshot. In this regard, AI-derived models, particularly when coupled with MD simulations, are well positioned to capture aspects of the dynamic behavior that underpins the distinctive gating kinetics of hERG. Continued development and integration of these approaches may ultimately provide deeper mechanistic insight and improve our ability to rationally target this clinically important channel.

## Methods

### Production of hERG Models

To model the hERG channel, we deployed the novel protein-folding software Chai-1 ^16^ using the Web server (https://lab.chaidiscovery.com/). To model the core channel, we used a truncated amino acid sequence of hERG, containing the trans-membrane domain (residues 400–718). We also ran a model for the entire hERG protein (residues 1–1159), and used previously published^17^ predictions of the core hERG channel with either the synthetic cannabinoid agonist 5F-AKB48 or the antiarrhythmic drug E-4031. For all models we used the default settings, with no MSAs and restraints applied, apart from one run where a MMseq2MSA was applied. In parallel, we used SwissModel (https://swissmodel.expasy.org/)^53^ to produce a tetrameric model of hERG residues 401–718 using the rat EAG closed structure (PDB: 5K7L).^9^ Alongside these, we obtained the recently published closed AF2 structure (trimmed to residues 398–719)^14^ and the experimentally determined open hERG structure (PDB: 9CHP; residues 398–709),^6^ Visual analysis of the channels was performed using PyMOL (Schrödinger LLC).

### Atomistic MD Simulations

MD simulations were conducted to assess the stability of our chosen Chai-1 hERG model. Proteins were described using the CHARMM36m force field.^54^ Each hERG model was placed into a lipid bilayer consisting 70% 1-palmitoyl-2-oleoyl-*sn*-glycero-3-phosphocholine (POPC), 20% cholesterol, and 10% palmitoylsphingomyelin (PSM). Membranes were solubilized with TIP3P water and neutralized with potassium chloride ions at concentration 0.15 M via CHARMM-GUI Membrane Builder.^55,56^ Protein side chains were all set to default protonation states.

The systems were minimized and equilibrated using the standard CHARMM-GUI protocol, with an additional 20 ns of NPT equilibration with xyz positional restraints of 50 kJ/mol/nm^2^ applied to the protein backbone. MD simulations were then conducted across each system for 1 μs with a total of 5 repeats. Simulations were run using GROMACS 2024.^57,58^ Simulations used a 2 fs time step in the NPT ensemble with the velocity-rescale thermostat and c-rescale pressure coupling. The results of the MD simulations were analyzed using built-in GROMACS tools and in-house scripts (see https://github.com/robincorey/hERG_closed_MD/). Visual analysis was performed using PyMOL (Schrödinger LLC) and VMD.^59^

### Analysis of Models

To help characterize the conformational state of the Chai-1 predicted hERG structures and post-MD snapshots (1 μs), the program HOLE was used to measure pore radius, using a Monte Carlo simulated annealing procedure to detect an optimum route through a channel.^60^ MD simulations were additionally analyzed using the CHAP package.^27^ Plots were generated using default CHAP scripts, adapted to set manual x and y limits. Visual analysis of the HOLE profiles was performed using PyMOL (Schrödinger LLC).

Interchain distances were calculated using MDAnalysis (2.10.0).^61,62^ For the pore analysis, hERG residues S624, Y652, and F656 were chosen, and the minimum heavy-atom distance between equivalent residues in diagonal protomers of the heterotetrameric complex was determined. For the VSD analysis, angles were calculated using MDAnalysis from Cα atom coordinates. For each chain, vectors were defined between residues 518–540 and 550–575, and the angle between the vectors was determined from the normalized dot product.

### Coarse-Grained MD Simulations

Coarse-grained molecular dynamics simulations were built for each of the hERG models. Protein atoms were converted to the CG Martini 3 force field^30^ using the martinize method.^63^ An elastic network was applied, comprising bonds of 500 kJ mol^–1^ nm^–2^ between all protein backbone beads within 0.9 nm. Proteins were built into membranes composed of 40% POPC (palmitoyl oleyl phosphatidylcholine), 10% POPE (palmitoyl oleyl phosphatidylethanolamine), 20% DPCE (ceramide), 20% cholesterol, 5% DIPC (dilinoleoylphosphatidylcholine), and 5% POPS (palmitoyl oleyl phosphatidylserine), using the insane method.^64^ All systems were solvated with Martini waters and Na^+^ and Cl^–^ ions to a neutral charge and 0.15 M. Systems were minimized using the steepest descents method, followed by 1 ns equilibration with 5 fs time steps, then by 100 ns equilibration with 20 fs time steps, before 5 × 10 μs production simulations using 20 fs time steps, all in the NPT ensemble at 323 K with the V-rescale thermostat (τ = 1.0 ps) and semi-isotropic Parrinello–Rahman pressure coupling at 1 bar (τ = 12.0 ps). The reaction-field method was used to model long-range electrostatic interactions. Bond lengths were constrained to the equilibrium values using the LINCS algorithm. Density analysis was performed using the VolMap tool of VMD, with the default settings.^59^ Lipid binding sites and lipid–residue interactions were determined using the PyLipID package, which provides both occupancy and residence time data.^31^ Reported occupancy and residence time values are analyzed using the total simulation time. Simulations were run using GROMACS 2024.^57,58^

## Supplementary Material



## Data Availability

All Chai-1 models and confidence metrics, along with pre and post-MD snapshots, are available for download from the OSF (https://osf.io/gbejy/). Scripts for analysis of Chai-1 models and snapshots from MD simulations are available on GitHub (https://github.com/robincorey/hERG_closed_MD/).

## Notes

Figure S1: Chai-1 pLDDT for all default Chai-1 models Figure S2: Alignment of default 5 Chai-1 models to the previously published AF2 closed structure Figure S3: Alignment of default 5 Chai-1 models to a homology model Figure S4: Alignment of default 5 Chai-1 models to open hERG structure Figure S5: All 5 default Chai-1 models compared to the previously published AF2 model Figure S6: View of hERG channel from Chai-1 predictions both with and without the E-4031 molecule. Figure S7: Alignment of MSA-enabled Chai-1 models to AF2 closed structure Figure S8: Alignment of MSA-enabled Chai-1 models to a homology model Figure S9: Alignment of MSA-enabled Chai-1 models to open hERG structure Figure S10: Backbone RMSDs for the entire hERG channel across the atomistic MD simulations Figure S11: Modeling the full-length Chai-1 monomer. Figure S12: Pore distances at S624 from MD Figure S13: Pore distances at Y652 from MD Figure S14: Water analysis in the hERG channel Figure S15: Interactions of ceramide with hERG from coarse-grained MD. Supplementary Table S1: Chai-1 metrics and structural metrics for the 5 primary Chai-1 models (PDF)
