# Supplementary material for: Integrating AI and Molecular Modeling for Structural Prediction of a Closed-Pore State of the hERG Channel
Source: J Chem Inf Model. 2026 Aug 10;66(16):10227–41. doi: 10.1021/acs.jcim.6c01219 (PMC13508769; doi:10.1021/acs.jcim.6c01219)
Supplement: Supplementary file 1 [file ci6c01219_si_001.pdf]

Supporting Information

Integrating AI and molecular modelling for structural prediction of a closed-pore state of the hERG channel

Catherine Upex[1], Thomas Osborne[2,3], Giovanni Biglino[1], Jules C. Hancox[2], Robin A Corey[3*]

[1] Bristol Medical School (THS), Level 7, Bristol Royal Infirmary, Upper Maudlin Street, Bristol, BS2 8HW
[2] Bristol Medical School (THS), Cardiovascular Research Laboratories, Biomedical Sciences Building, University Walk, Bristol, BS2 8HW
[3] School of Psychology and Neuroscience, Biomedical Sciences Building, University Walk, Bristol, BS8 1TD.

* author for correspondence: robin.corey@bristol.ac.uk

Supplementary figures:

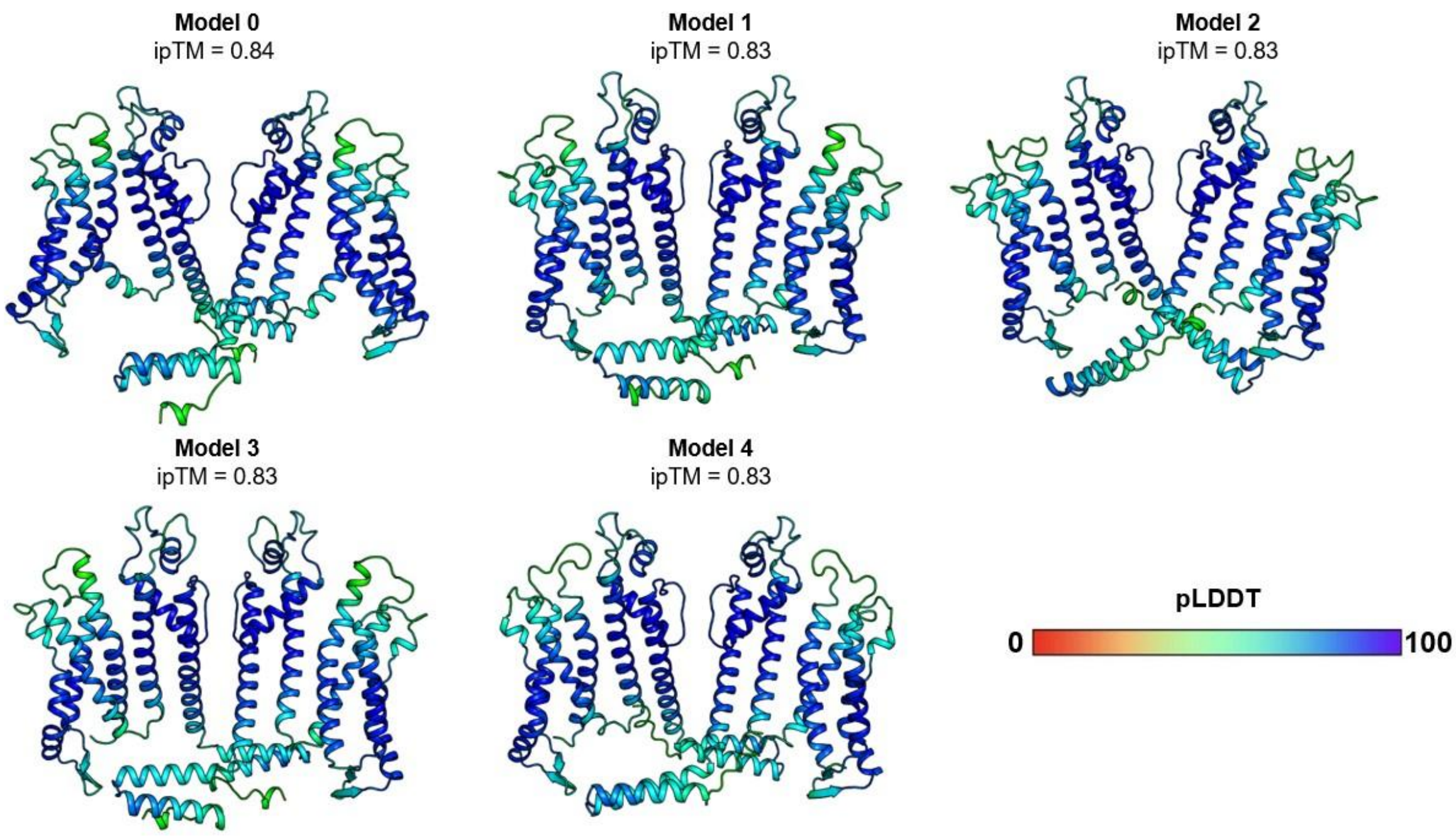

Figure S1: Chai-1 pLDDT for all default Chai-1 models. Two out of four subunits for each protein are shown for clarity, and chains are coloured by Chai-1 generated pLDDT score.

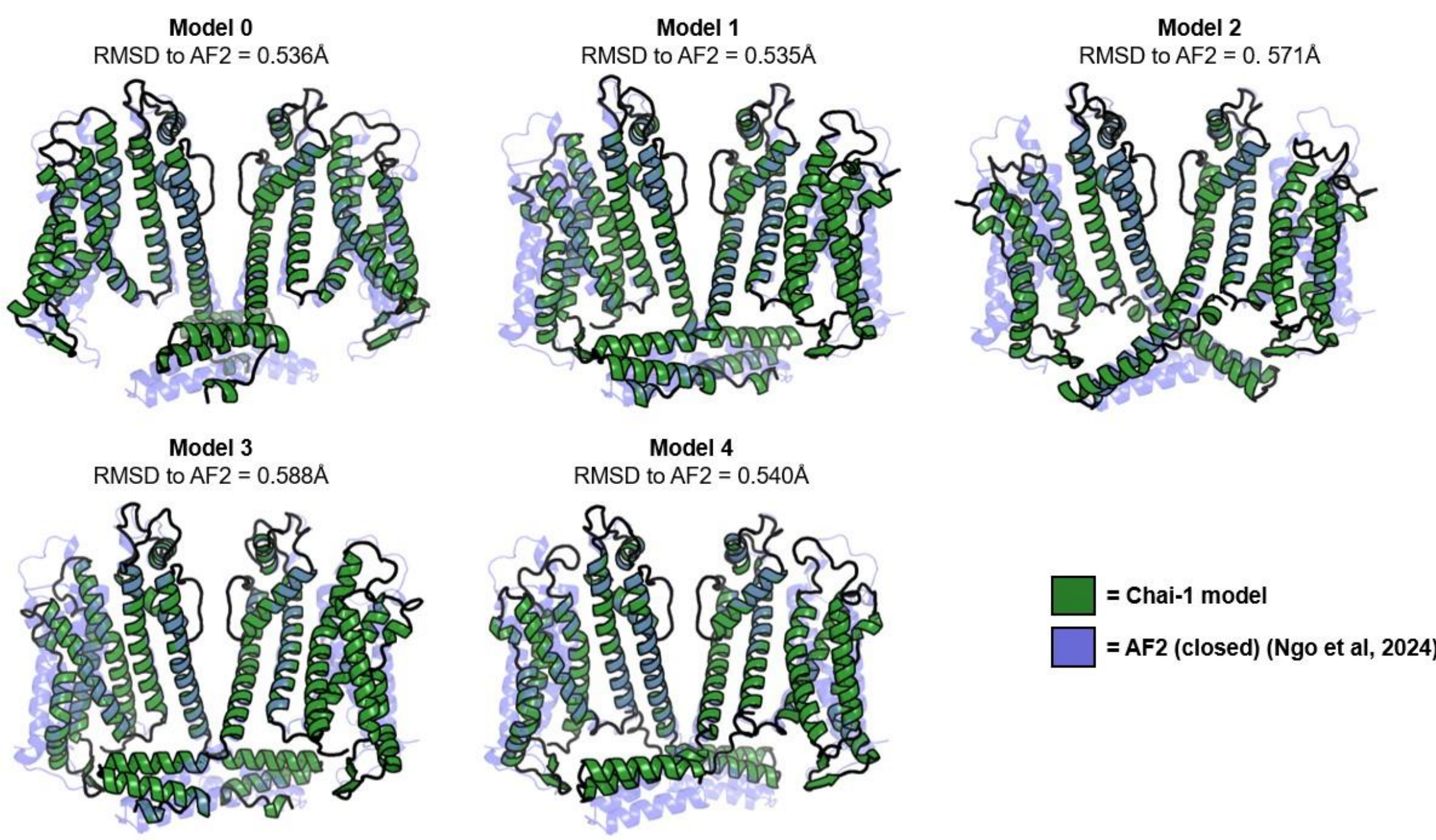

Figure S2: Alignment of default 5 Chai-1 models to the previously published AF2 closed structure. Two out of four subunits for each protein are shown for clarity, with Chai-1 models in green and AF2 model in transparent blue. Computed RMSDs are shown for each. RMSDs are run on the pore domain – residues 546-670 – as is also the same for Figures S3, S4, S7, S8 and S9.

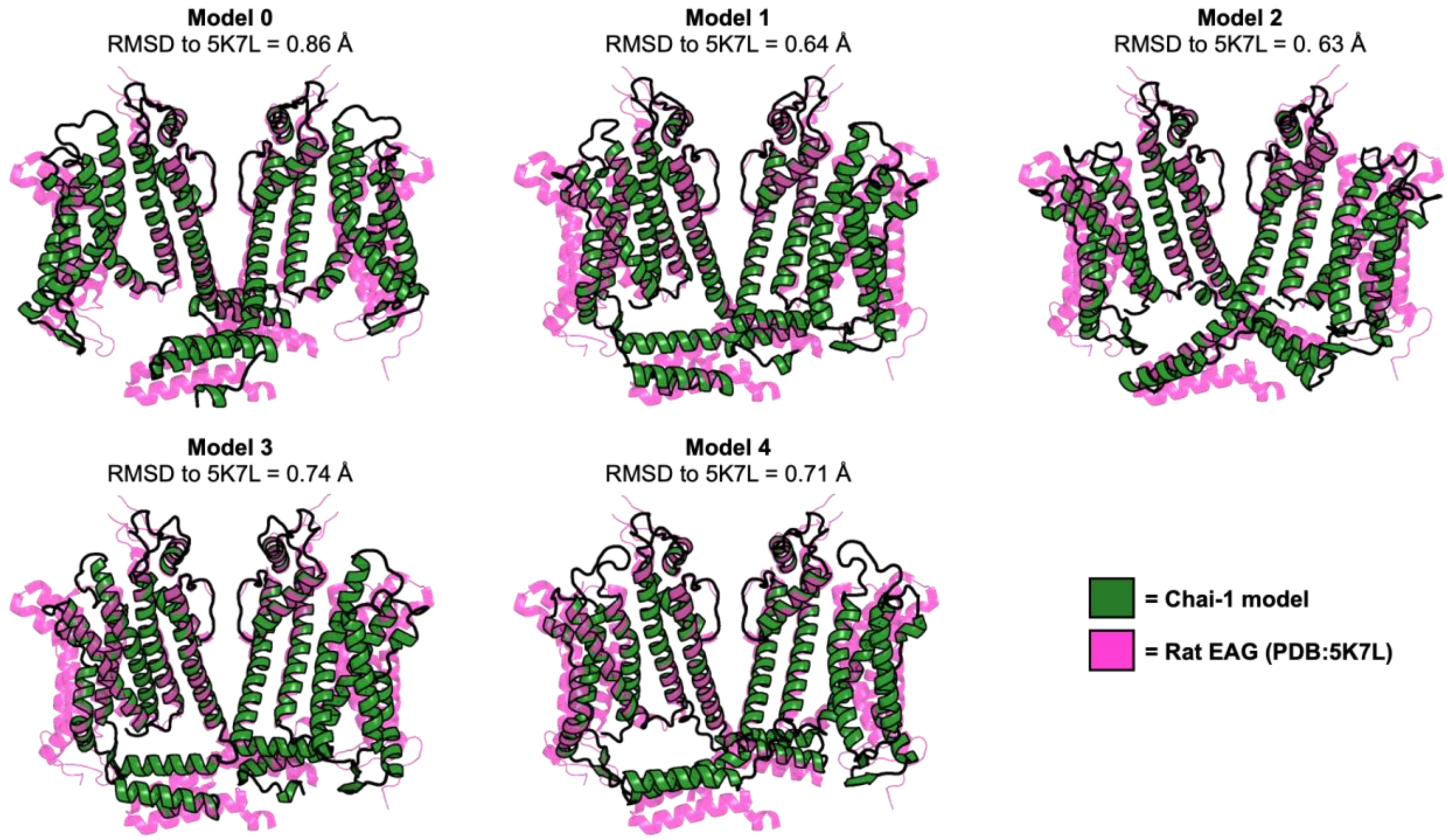

Figure S3: Alignment of default 5 Chai-1 models to the previously published *Rattus norvegicus* EAG channel. Two out of four subunits for each protein are shown for clarity, with Chai-1 models in green and EAG structure model in pink. Computed RMSDs are shown for each model.

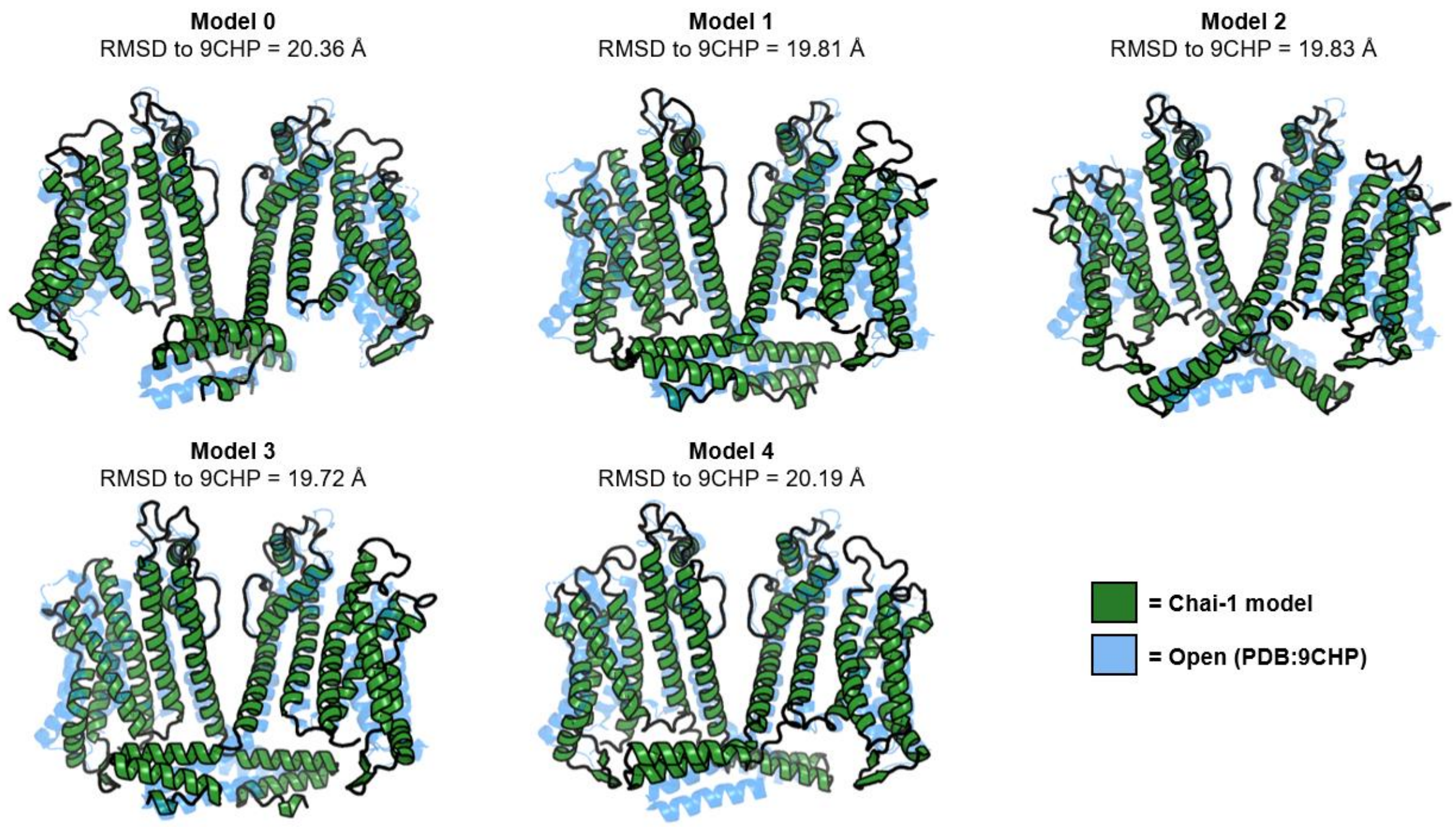

Figure S4: Alignment of default 5 Chai-1 models to the previously published experimentally-derived open hERG structure (PDB:9CHP). Two out of four subunits for each protein are shown for clarity, with Chai-1 models in green and open PDB in transparent light blue. Computed RMSDs are shown for each model.

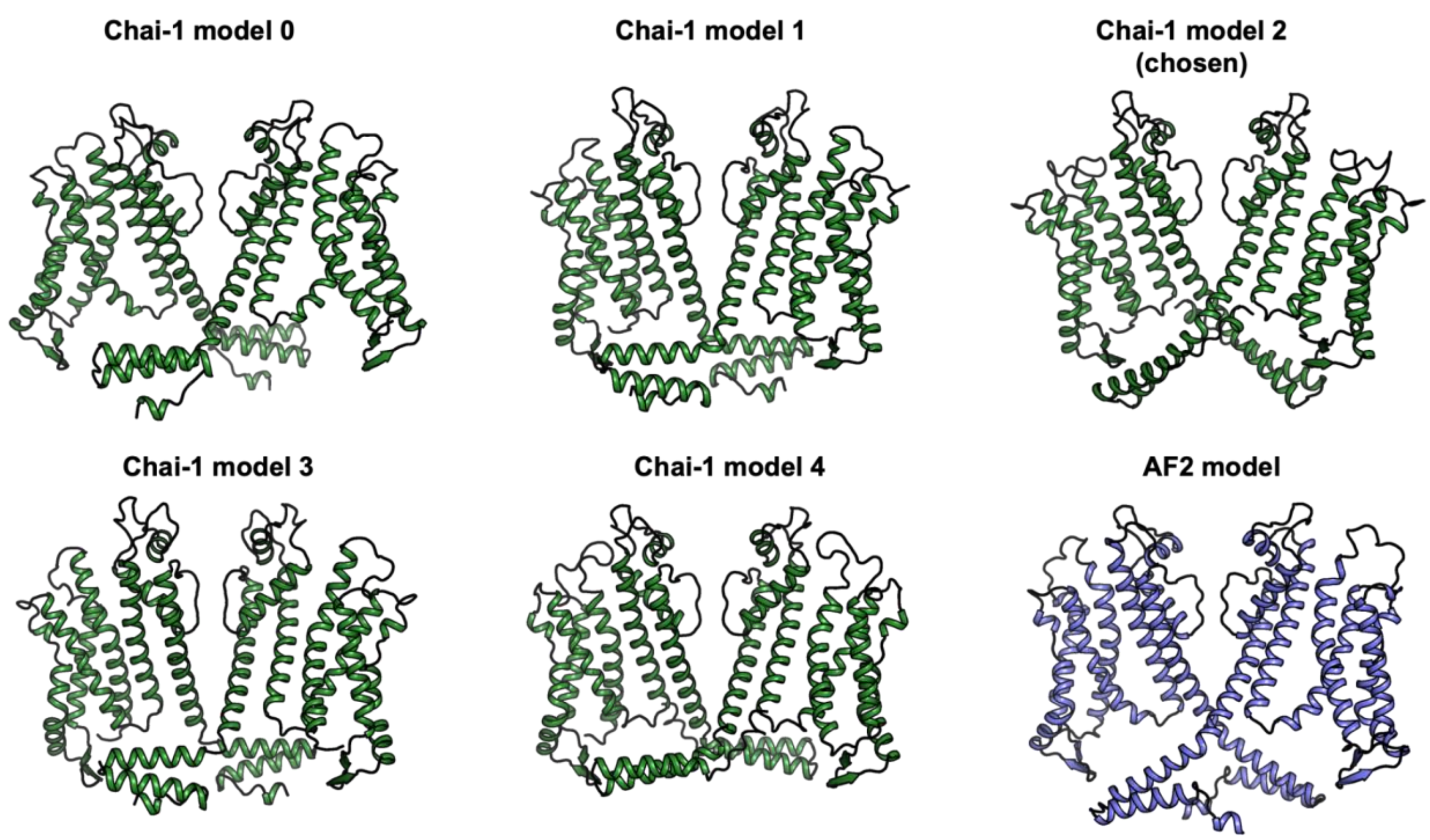

Figure S5: All 5 default Chai-1 models compared to the previously published AF2 model, highlighting the one chosen for downstream analysis. Two out of four subunits for each protein are shown for clarity.

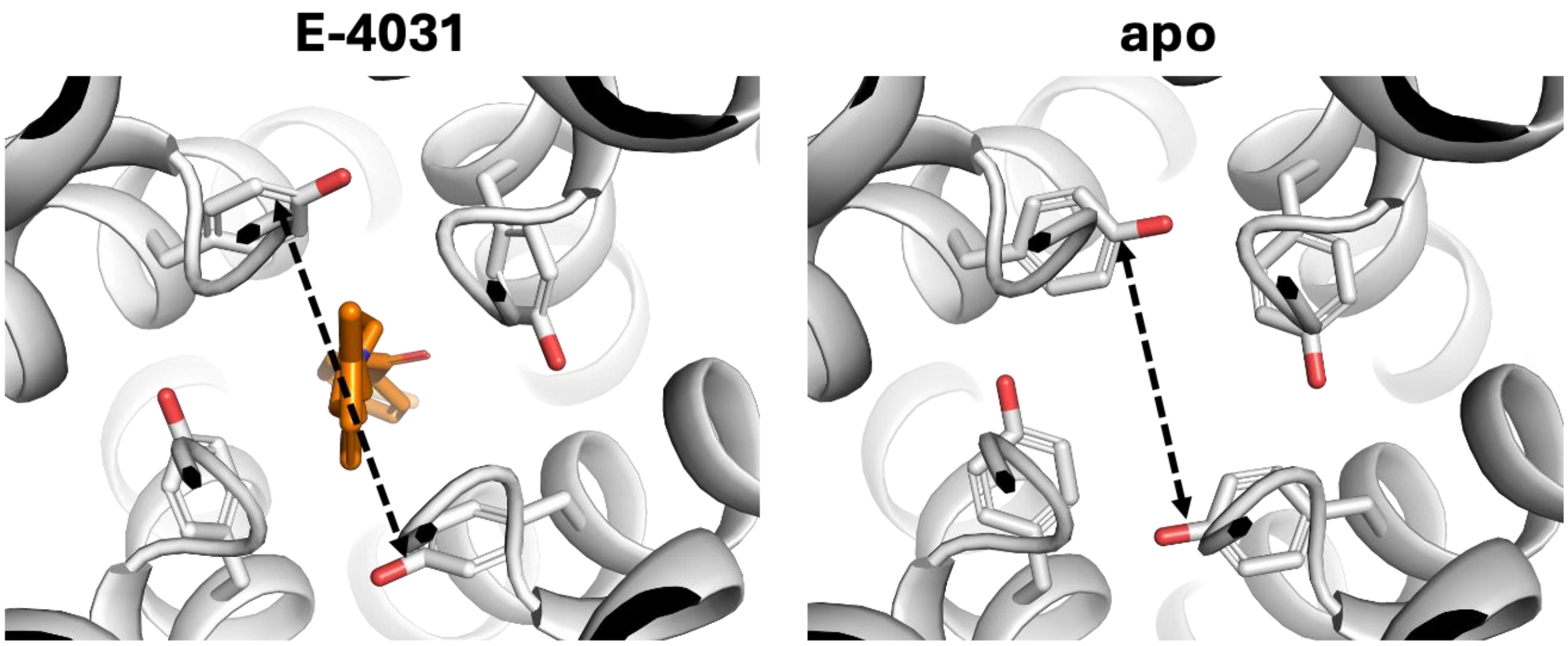

Figure S6: View of hERG channel from Chai-1 predictions both with and without the E-4031 molecule. The presence of the drug (orange sticks) in some cases causes the distance between opposing Y652 side chains to increase slightly (sticks). The dotted line is for illustrative purposes only.

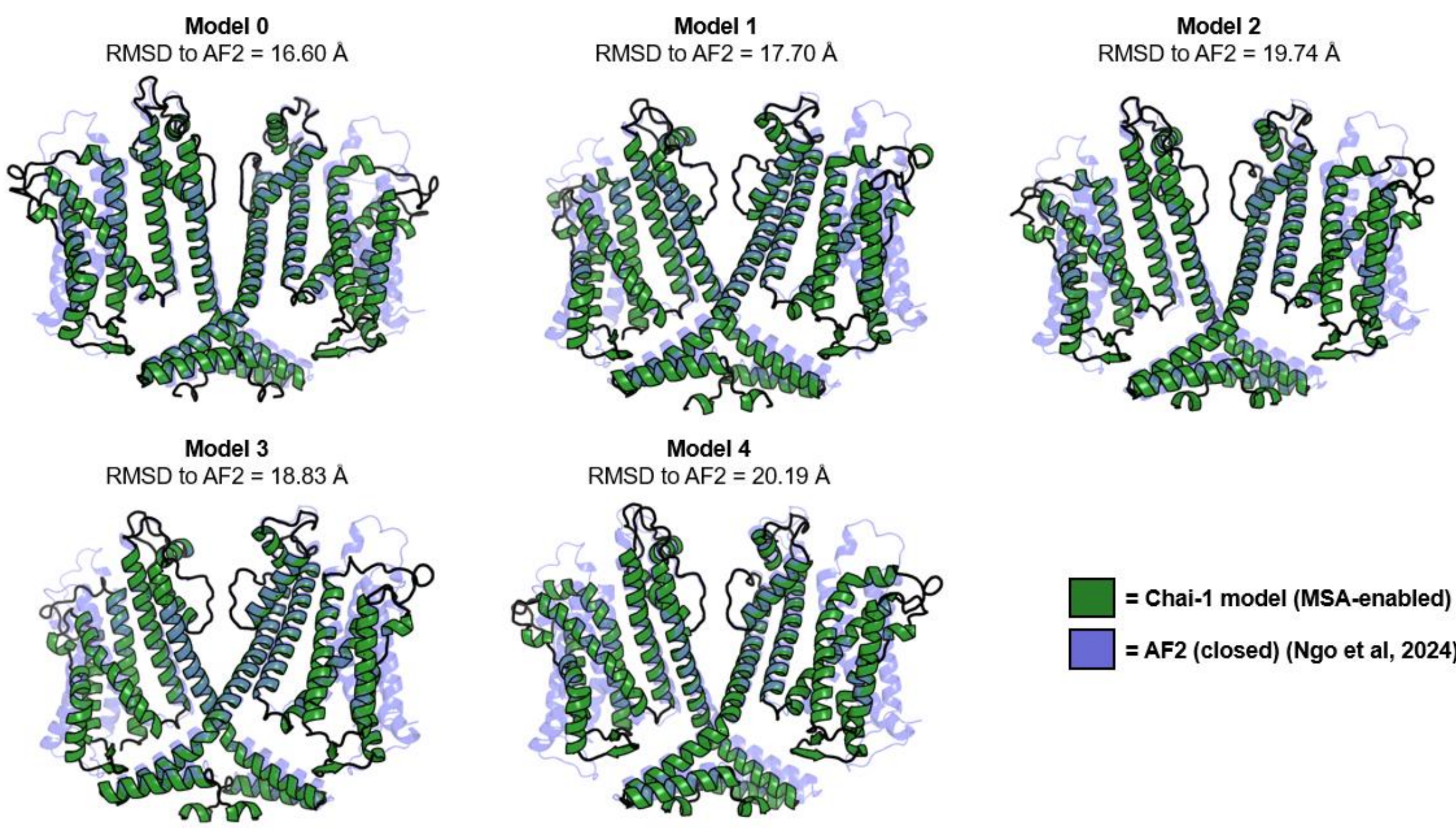

Figure S7: Alignment of MSA-enabled Chai-1 models to the previously published AF2 closed structure. Two out of four subunits for each protein are shown for clarity, with Chai-1 models in green and AF2 model in transparent blue. Computed RMSDs are shown for each model.

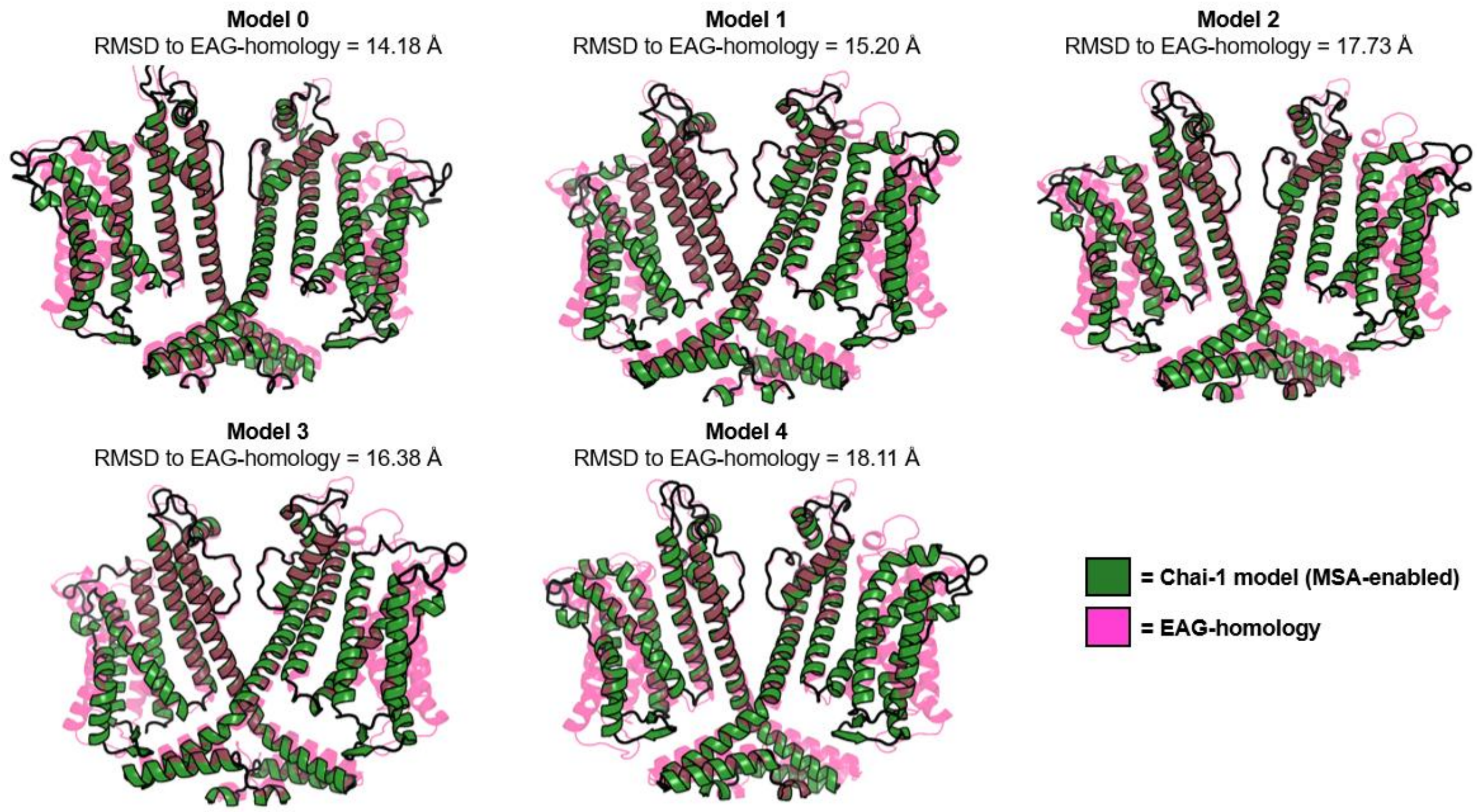

Figure S8: Alignment of MSA-enabled Chai-1 models to a homology model based on the previously published *Rattus norvegicus* EAG channel. Two out of four subunits for each protein are shown for clarity, with Chai-1 models in green and AF2 model in transparent blue. Computed RMSDs are shown for each model.

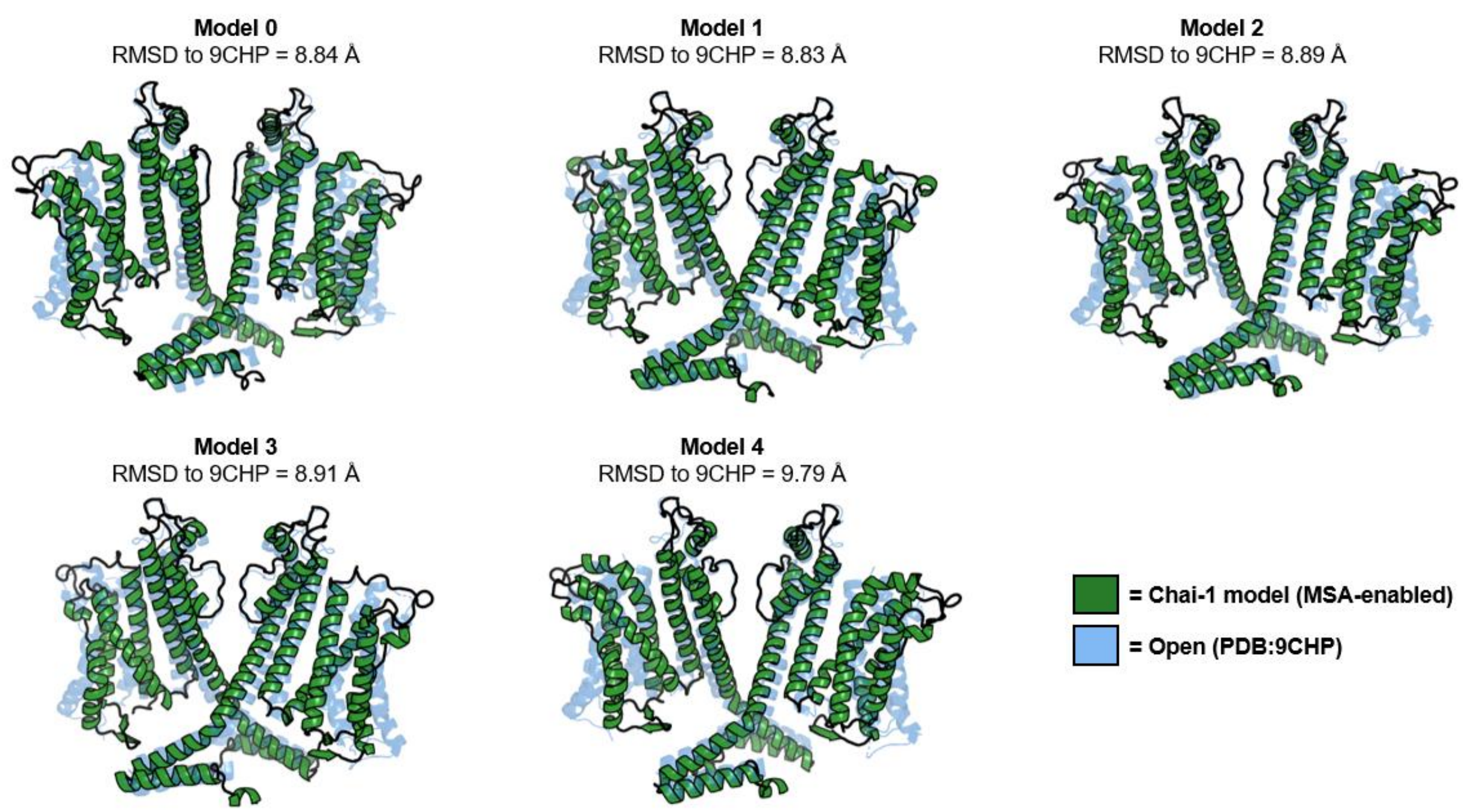

Figure S9: Alignment of MSA-enabled Chai-1 models to the previously published experimentally-derived open hERG structure (PDB:9CHP). Two out of four subunits for each protein are shown for clarity, with Chai-1 models in green and open PDB in transparent light blue. Computed RMSDs are shown for each model.

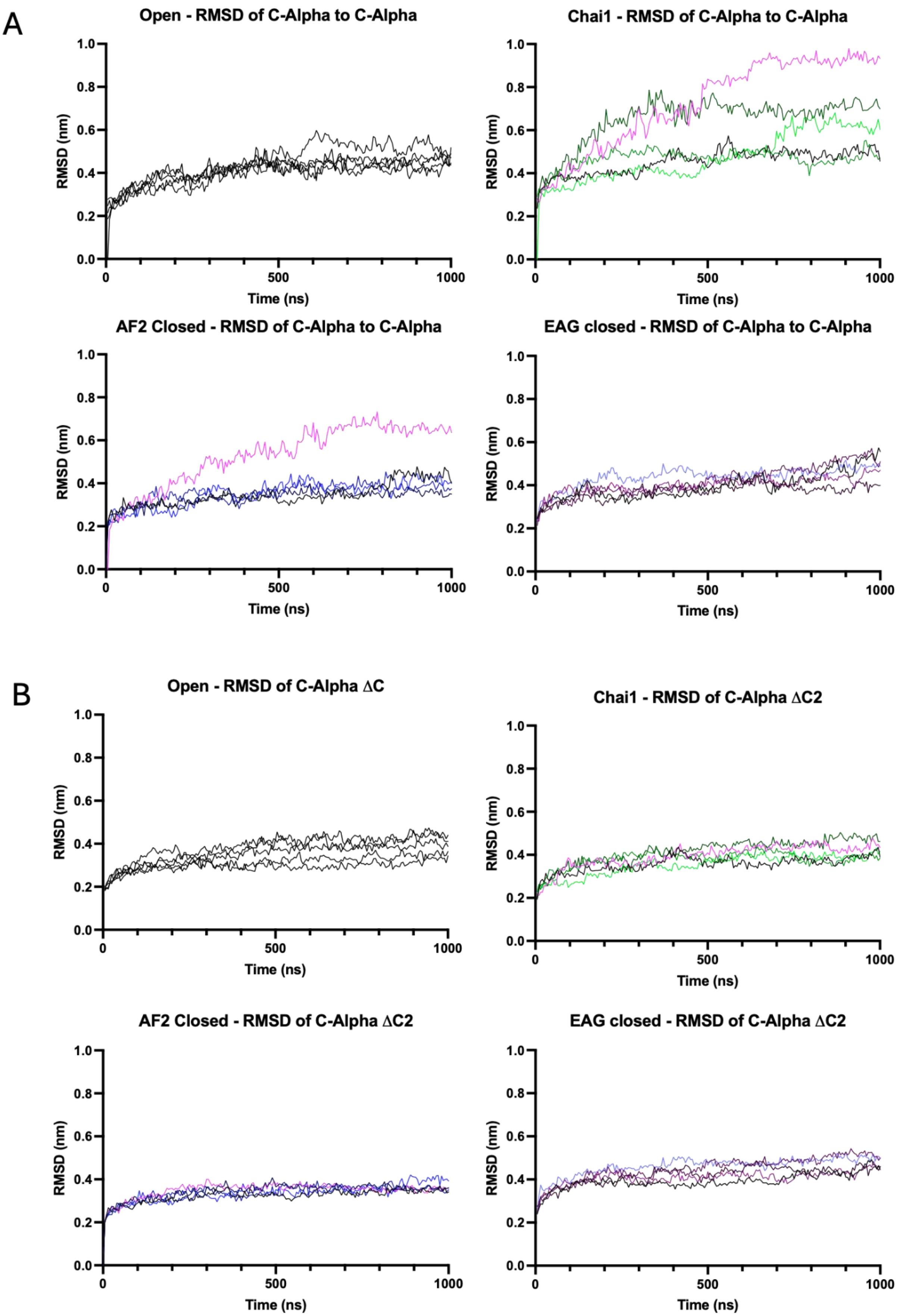
A
Open - RMSD of C-Alpha to C-Alpha
Chai1 - RMSD of C-Alpha to C-Alpha
AF2 Closed - RMSD of C-Alpha to C-Alpha
EAG closed - RMSD of C-Alpha to C-Alpha
B
Open - RMSD of C-Alpha ΔC
Chai1 - RMSD of C-Alpha ΔC2
AF2 Closed - RMSD of C-Alpha ΔC2
EAG closed - RMSD of C-Alpha ΔC2
RMSD (nm)
Time (ns)
0.0
0.2
0.4
0.6
0.8
1.0
0
500
1000

Figure S10: Backbone RMSDs for the entire hERG channel across the atomistic MD simulations for each of the hERG models. (A) RMSDs are for C-alpha atoms following a global fit across C-alpha atoms. Five MD repeats are shown for each system. Chai-1 and AF2 both have one repeat with a higher RMSD than the others (magenta). (B) RMSDs for C-alpha atoms with the C-linker removed from analysis (residues after 666).

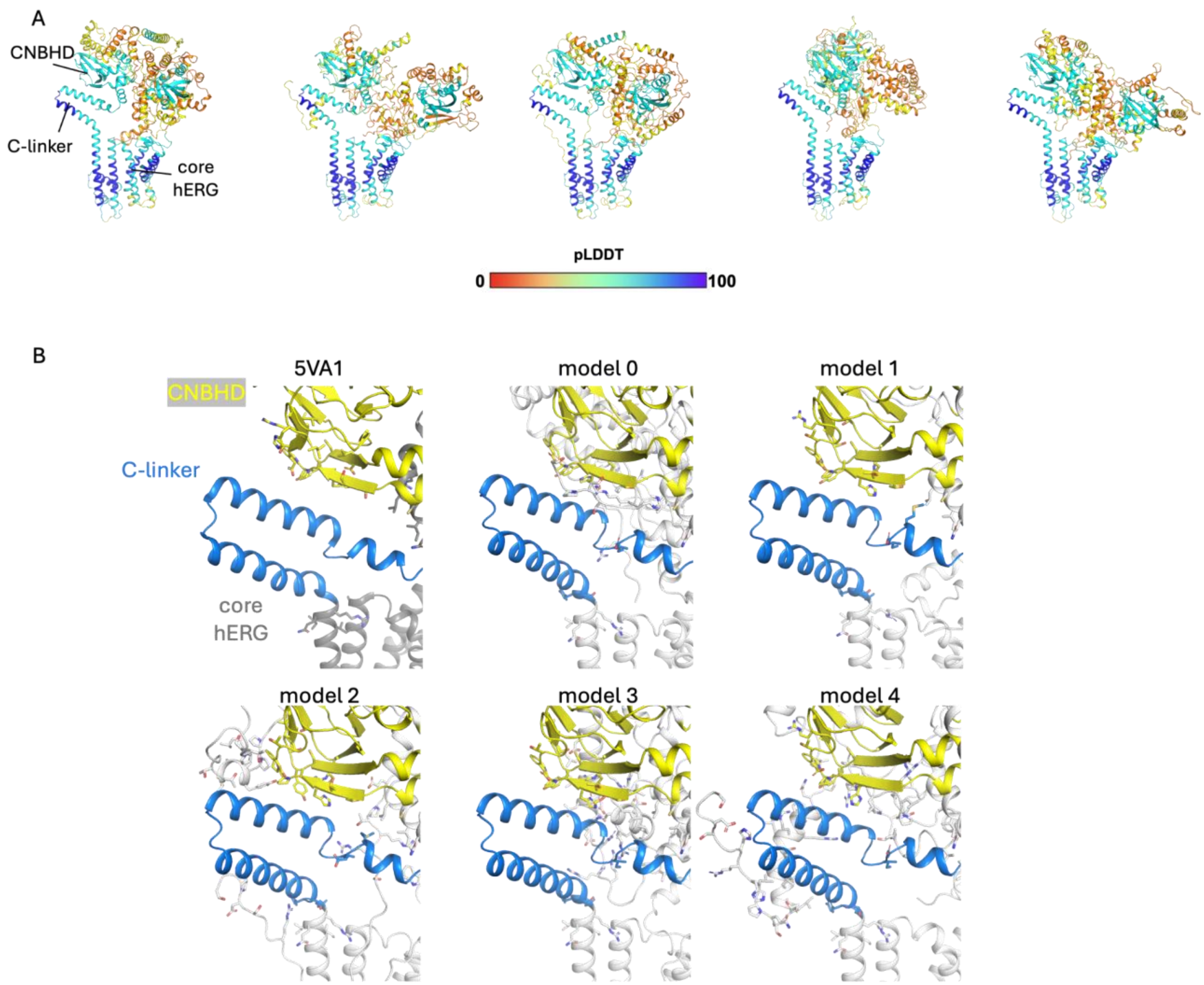

Figure S11: Modelling the full-length Chai-1 monomer. (A) Shown are the models produced by Chai-1 when modelling a full-length monomer chain. Note the very low pLDDT outside of the core region. The core region, the C-linker, and the CNBHD are highlighted for model 0. (B) A close up of the C-linker/CNBHD region, showing the variance of the models in this region in relation to 5VA1. Residues from hERG that are interacting with the c-linker are shown as sticks.

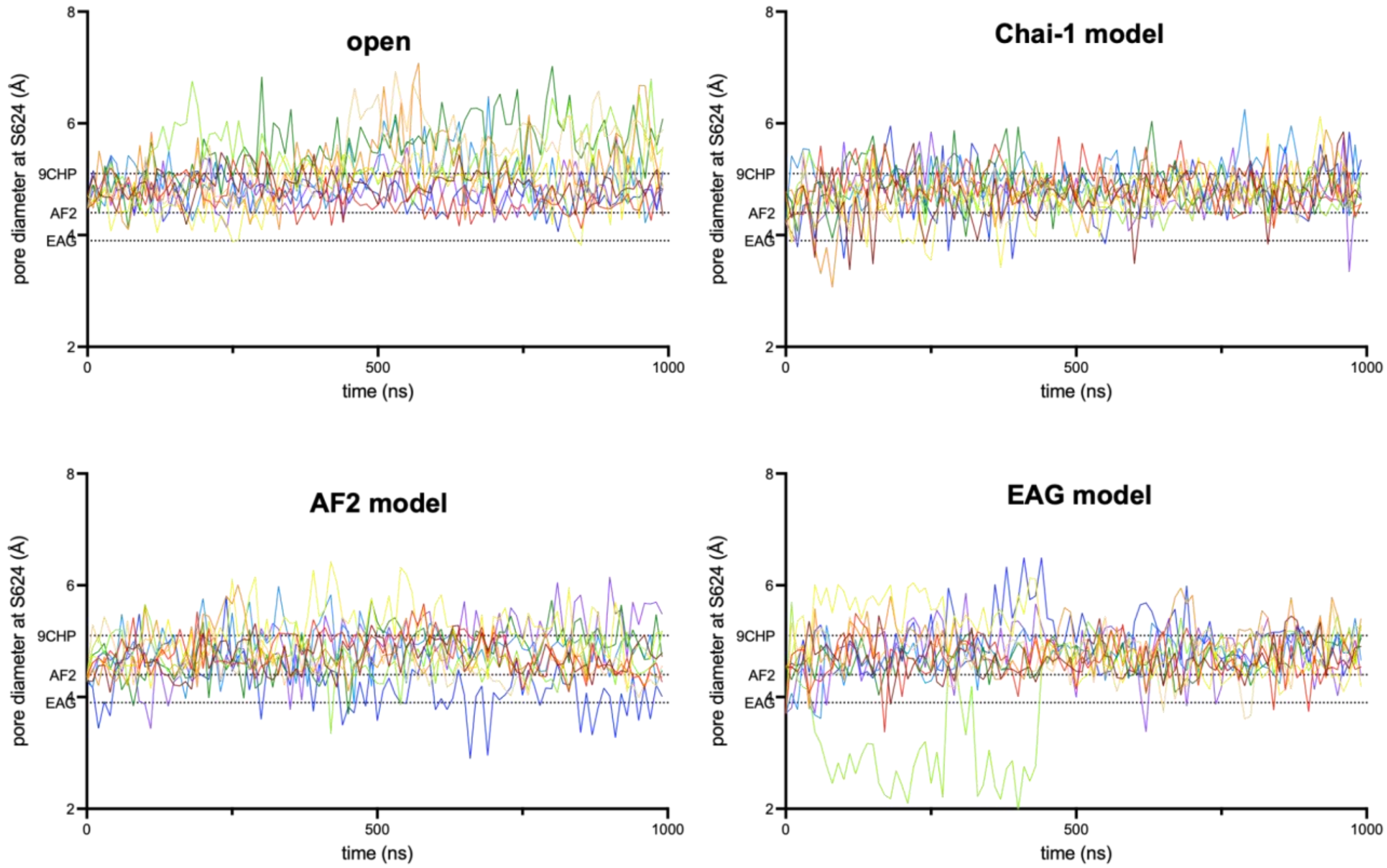

Figure S12: Pore distances at S624. As per Figure 4, distances between Ser624 residues from opposing subunits are plotted over time during 5 x 1000 ns atomistic MD simulations for the open structure, and Chai-1, AF2 closed, and EAG models. Two distances are shown for each system, corresponding to chains A-C and B-D. Distances measured in the input models are indicated by dotted lines.

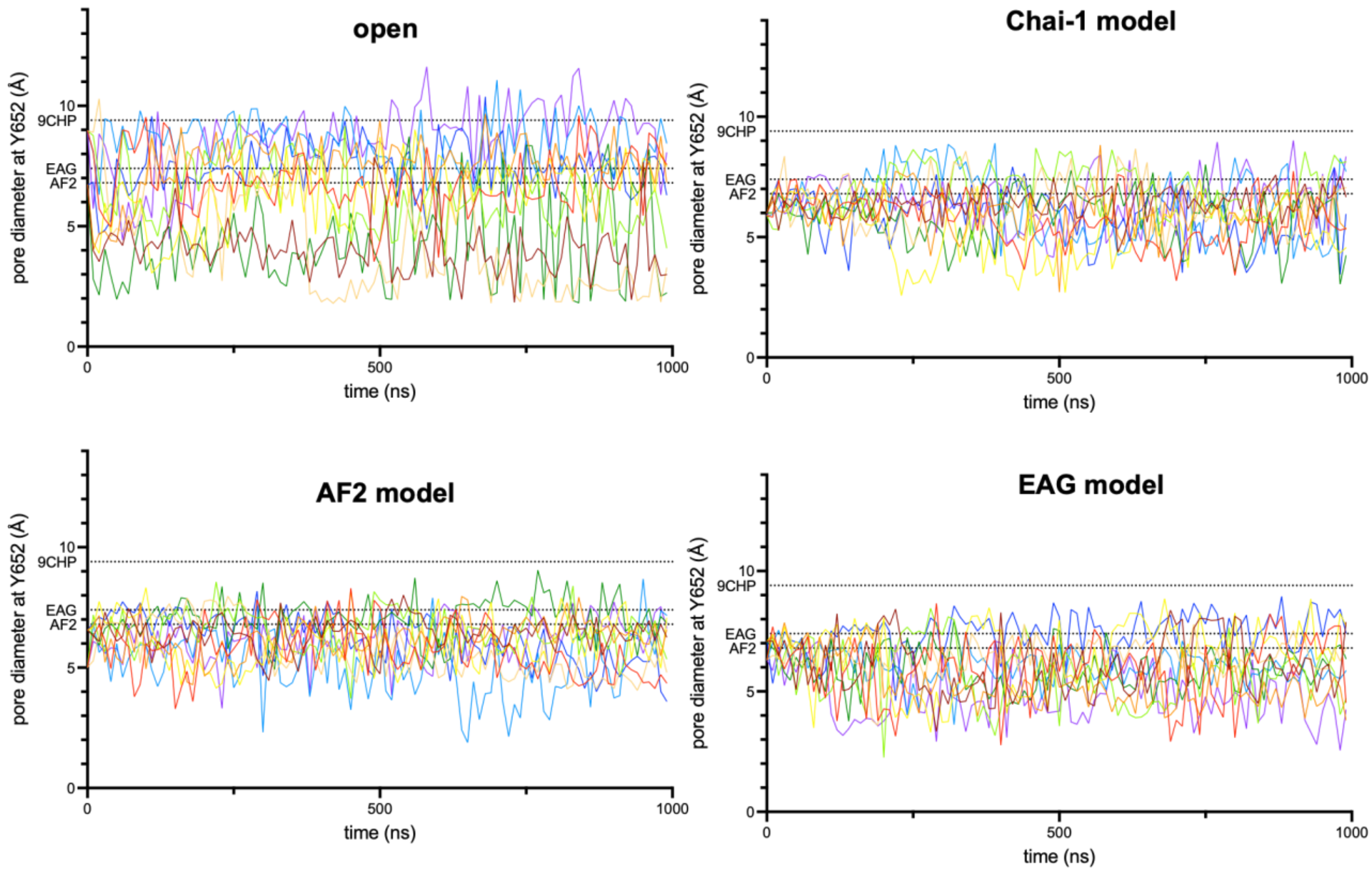

Figure S13: Pore distances at Y652. As per Figure S12 but for the pore diameter at Y652.

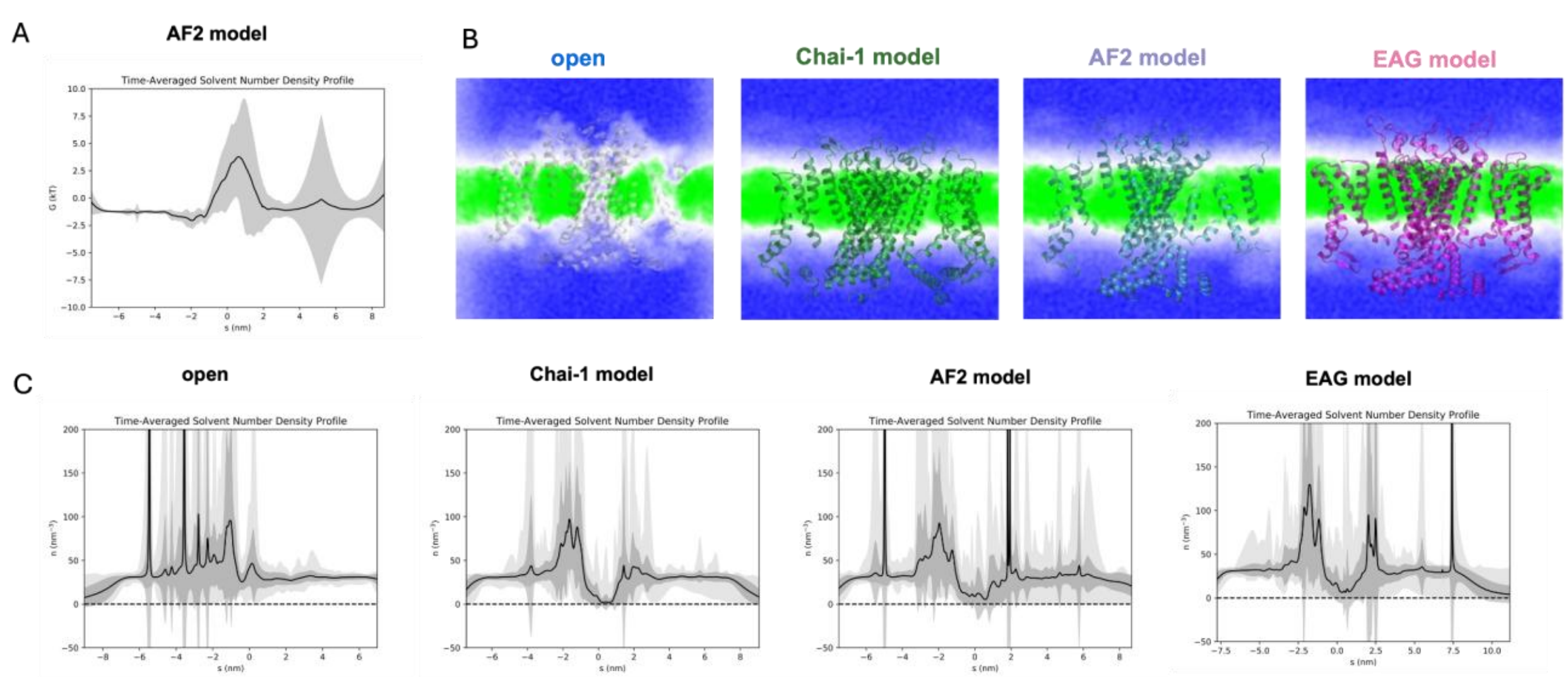

Figure S14: Water analysis in the hERG channels (A) As per Figure 5A, but for the AF2 model. The CHAP profile shows a similar barrier to solvation in the central pore. Note that we were unable to computate energy profiles for the EAG model, but do provide solvation profiles for all systems in panel C.

(B) Densities of waters (blue) and lipids (green) computed over 5 x 1 µs of MD simulation for each model using VMD. (C) Solvation profiles through the hERG channels computed over 5 x 1 µs of MD. The water density remains above ca. 25 for the open channel, whereas it dips to 0 in simulations of all 3 closed models.

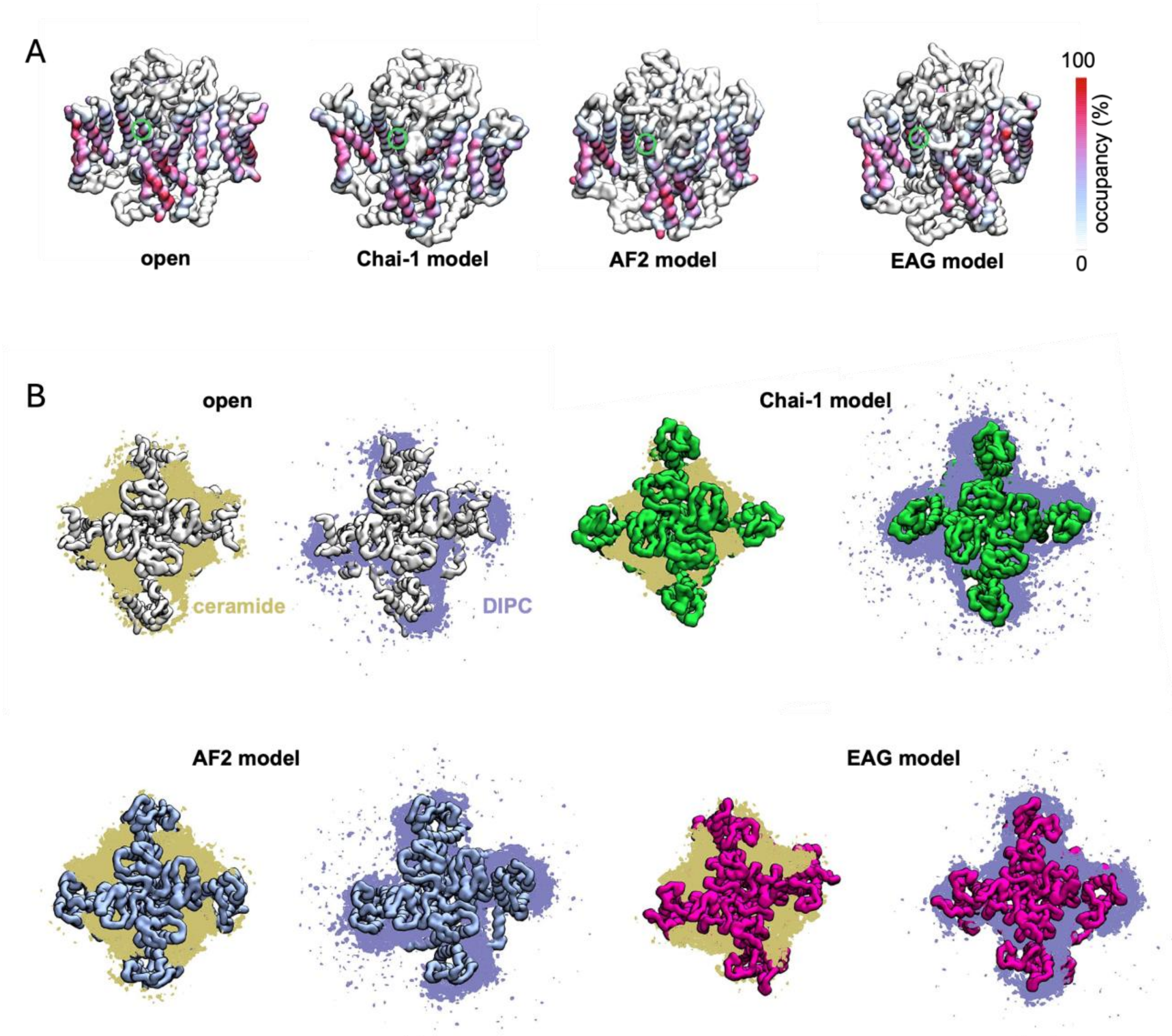

Figure S15: Interactions of ceramide in relation to each of the hERG models based on 5 x 10 µs of coarse-grained MD. (A) PyLipID-derived occupancies for ceramide, projected on the hERG structures. M651 position is denoted with a green circle. (B) Densities computed using VMD VolMap. Gold relates to ceramide (DPCE) density. Blue relates to DIPC (dilinoleoyl PC) density.

Chai-1 model	pLDDT	ipTM	RMSD to AF2	RMSD to EAG closed
0	83.91	0.836	0.54	0.86
1	82.97	0.826	0.54	0.64
2	83.45	0.830	0.57	0.63
3	82.87	0.825	0.59	0.74
4	83.45	0.830	0.54	0.71

Supplementary Table S1: Major metrics from Chai-1 model and summary of RMSDs to reference structures for the primary 5 Chai-1 models.
